# SC3-seq: a method for highly parallel and quantitative measurement of single-cell gene expression

**DOI:** 10.1093/nar/gkv134

**Published:** 2015-02-26

**Authors:** Tomonori Nakamura, Yukihiro Yabuta, Ikuhiro Okamoto, Shinya Aramaki, Shihori Yokobayashi, Kazuki Kurimoto, Kiyotoshi Sekiguchi, Masato Nakagawa, Takuya Yamamoto, Mitinori Saitou

**Affiliations:** 1 Department of Anatomy and Cell Biology, Graduate School of Medicine, Kyoto University, Yoshida-Konoe-cho, Sakyo-ku, Kyoto 606-8501, Japan; 2 JST, ERATO, Yoshida-Konoe-cho, Sakyo-ku, Kyoto 606-8501, Japan; 3 Center for iPS Cell Research and Application, Kyoto University, 53 Kawahara-cho, Shogoin Yoshida, Sakyo-ku, Kyoto 606-8507, Japan; 4 Institute for Protein Research, Osaka University, Osaka 565-0871, Japan; 5 Institute for Integrated Cell-Material Sciences, Kyoto University, Yoshida-Ushinomiya-cho, Sakyo-ku, Kyoto 606-8501, Japan

## Abstract

Single-cell mRNA sequencing (RNA-seq) methods have undergone rapid development in recent years, and transcriptome analysis of relevant cell populations at single-cell resolution has become a key research area of biomedical sciences. We here present single-cell mRNA 3-prime end sequencing (SC3-seq), a practical methodology based on PCR amplification followed by 3-prime-end enrichment for highly quantitative, parallel and cost-effective measurement of gene expression in single cells. The SC3-seq allows excellent quantitative measurement of mRNAs ranging from the 10,000-cell to 1-cell level, and accordingly, allows an accurate estimate of the transcript levels by a regression of the read counts of spike-in RNAs with defined copy numbers. The SC3-seq has clear advantages over other typical single-cell RNA-seq methodologies for the quantitative measurement of transcript levels and at a sequence depth required for the saturation of transcript detection. The SC3-seq distinguishes four distinct cell types in the peri-implantation mouse blastocysts. Furthermore, the SC3-seq reveals the heterogeneity in human-induced pluripotent stem cells (hiPSCs) cultured under on-feeder as well as feeder-free conditions, demonstrating a more homogeneous property of the feeder-free hiPSCs. We propose that SC3-seq might be used as a powerful strategy for single-cell transcriptome analysis in a broad range of investigations in biomedical sciences.

## INTRODUCTION

Quantitative transcriptome analysis at single-cell resolution is becoming an increasingly important area of biomedical sciences, including in the research fields of developmental/stem cell/cancer biology, and is providing a foundation for understanding the regulation of gene expression in single cells in physiology or diseased states at a systems level (1,2). Currently, single-cell mRNAs/cDNAs need to be amplified prior to global quantitative assessments. There have been two major approaches to the amplification of genes expressed in single cells: methods involving exponential amplification by polymerase chain reaction (PCR) and methods involving linear amplification by T7 RNA polymerase (3,4). The methods involving exponential amplification have higher amplification efficiency, greater methodological simplicity and higher stability of the amplified products, which allows an examination of the amplification quality prior to global measurements/repeated assessment of the same single-cell transcriptomes. Accordingly, these methods have been more prevalently used for single-cell transcriptome analyses in practical experimental settings (1,2,5,6).

To ensure quantitative/representative amplification of single-cell cDNAs, one of the original methods that applied amplified cDNAs to global analyses using high-density oligonucleotide microarrays restricted the length of the first-strand cDNAs to, on average, ∼700 base pairs (bp) from the 3-prime ends [transcription termination sites (TTSs)] of mRNAs, by a short (5 min) reverse transcription (7,8). Subsequently, this amplification method has been modified so that longer first-strand cDNAs including full-length cDNAs are synthesized and the amplified products can be applied to RNA sequencing (RNA-seq) analyses (9–11). As an alternative approach, single-cell cDNA amplification protocols that enrich full-length cDNAs using ‘template switching’ technology have also been applied to RNA-seq analyses (12,13). In addition, to facilitate more absolute quantification of transcript levels, methodologies that tag the 5-prime [transcription start sites (TSSs)] or 3-prime ends (TTSs) of the first-strand cDNAs/mRNAs in single cells with unique molecular identifiers (UMIs) and amplify cDNAs by exponential or linear amplification for RNA-seq analyses have been reported (14–18). Finally, it has become possible to simultaneously analyze the transcriptomes of thousands of single cells by exploiting the barcodes that distinguish these individual cells and by using microfluidics to automatically capture and process them in large numbers; this, in turn, should open a pathway to clarification of the comprehensive cellular decomposition of complex tissues/organs (19,20).

Although the technology for single-cell transcriptome analysis has thus been expanding rapidly, there remain a number of issues that deserve careful consideration. For example, synthesis of full-length cDNAs by reverse transcription would not be an efficient process (9–11), template switching technology would harbor inherent/stochastic errors (12,13) and amplification of full-length cDNAs, especially those with longer length, by PCR would be susceptible to amplification bias (21). It should also be noted that accurate quantification of expression levels by UMIs requires a massive depth of sequence reads (17,20). Based on these facts/considerations, we reason that amplification and sequencing of the 3-prime ends of single-cell cDNAs would provide more precise quantification of single-cell cDNAs with a relatively small depth of sequence reads, allowing a highly parallel analysis of a large number of single cells in a broader range of more practical experimental settings. We here report single-cell mRNA 3-prime end sequencing (SC3-seq), a simple and practical methodology for highly parallel and quantitative measurement of genes expressed in single cells.

## MATERIALS AND METHODS

### Isolation of RNA/single cells for the SC3-seq analysis

All the animal experiments were performed under the ethical guidelines of Kyoto University. The mouse embryonic stem cell (mESC) line BVSC R8 was cultured as reported previously (22), and total RNAs from the line were extracted using an RNeasy mini kit [Qiagen (74104), Hilden, Germany] according to the manufacturer's instructions. The isolated RNAs were serially diluted by double-distilled water (DDW) to concentrations of 250 ng/μl, 25 ng/μl, 2.5 ng/μl, 250 pg/μl and 25 pg/μl for use in evaluation of the quantitative performance of the SC3-seq.

For isolating mouse blastocysts, C57BL/6 mice were mated and noon of the day when a copulation plug was identified was designated as embryonic day (E) 0.5. At E4.5, peri-implantation blastocysts were flushed from the uteri by KSOM [Merck Millipore (MR-020P-5D), Darmstadt, Germany], and then they were bisected into a polar part containing an inner cell mass (ICM) and polar trophectoderm (pTE) and a mural part containing mural TE (mTE) by a glass needle under a dissection microscope [Leica Microsystems (M80), Wetzlar, Germany]. Each fragment was incubated with 0.25% trypsin/phosphate buffered saline (PBS) [Sigma-Aldrich (T4799), St. Louis, MO, USA] for around 10 min at 37°C, then dissociated into single cells by repeated pipetting and dispersed in 0.1 mg/ml of PVA/PBS [Sigma-Aldrich (P8136)] in preparation for the SC3-seq analysis.

The experimental protocols dealing with human subjects were approved by the institutional review board (Ethics Committee, Graduate School of Medicine, Kyoto University), with written informed consent provided by each donor. For the analysis of human-induced pluripotent stem cells (hiPSCs), the two iPSC lines, 585A1 and 585B1 (23), were cultured either under a conventional culture condition [DMEM/F12 [Life Technologies (11330-32), Carlsbad, CA, USA] supplemented with 20% (vol/vol) Knockout Serum Replacement [KSR; Life Technologies (10828-028)], 1% (vol/vol) GlutaMax [Life Technologies (35050-061)], 0.1-mM nonessential amino acids [Life Technologies (11140-050)], 4-ng/ml recombinant human bFGF [Wako Pure Chemical Industries (064-04541), Osaka, Japan] and 0.1-mM 2-mercaptoethanol [Sigma-Aldrich (M3148)]] on the SNL feeder cells (24) or under a feeder-free condition as reported previously (25,26). For the isolation of single hiPSCs cultured with the feeders, the culture was first treated with CTK solutions [0.25% Trypsin [Life Technologies (15090-046)], 0.1-mg/ml Collagenase IV [Life Technologies (17104-019)], 1-mM CaCl_2_ [Nacalai Tesque (06729-55)]] (27) for the removal of the feeder cells, then dissociated into single cells using Accutase [Innovative Cell Technologies, San Diego, CA, USA]. For the preparation of single cells from a feeder-free system, the cells were dissociated into single cells with 0.5 × TrypLE Select [TrypLE Select [Life Technologies (12563011)] diluted 1:1 with 0.5-mM ethylenediaminetetraacetic acid/PBS] (25). Dissociated single hiPSCs were transferred several times into a pick-up medium consisting of 1% KSR/PBS containing 10 μM of the ROCK inhibitor Y-27632 [Wako Pure Chemical Industries (257-00511)] (24) in preparation for the SC3-seq analysis.

### cDNA synthesis and amplification for the SC3-seq analysis

cDNA synthesis and amplification from isolated RNAs/single cells were performed essentially as reported previously (7,8), except that the Qiagen RNase inhibitor [0.4 U/sample, Qiagen (129916)] and the Porcine Liver RNase inhibitor [0.4 U/sample, Takara Bio (2311A)] were used and that the spike-in RNAs developed by the External RNA Controls Consortium [ERCC; Life Technologies (4456740)] were used and different numbers of PCR cycles were employed for amplification depending on the amounts of starting total RNAs (total RNA 100 ng: seven cycles; 10 ng: 11 cycles; 1 ng: 14 cycles; 100 pg: 17 cycles; 10 pg: 20 cycles). A total of 62 316 or 12 463 copies of the ERCC spike-in RNAs were added to the Lysis buffer (7) per 10 pg of total RNAs and single cells, respectively (see Supplementary Table S2 for the copy numbers of the ERCC spike-in RNAs). Prior to the construction of the SC3-seq library, the quality of the amplified cDNAs was evaluated by examining the Ct values of the quantitative real-time PCR (Q-PCR) of the ERCC spike-in RNAs (for mouse ESC total RNA dilution analysis: ERCC-00074, 9030 copies; ERCC-00004, 4515 copies; ERCC-00113, 2257 copies; ERCC-00136, 112.8 copies; ERCC-00042, 282.2 copies; ERCC-00095, 70.5 copies; ERCC-00019, 17.6 copies; and ERCC-00154: 4.4 copies; for the analyses of mouse pre-implantation embryos and hiPSCs: ERCC-00096: 1806 copies; ERCC-00171: 451.5 copies; ERCC-00111: 56.4 copies) and several endogenous genes (see Supplementary Table S3 for the primer list), and by examining the cDNA fragment proportion by LabChip GX [Perkin Elmer, Waltham, MA, USA] or Bioanalyzer 2100 [Agilent Technologies, Palo Alto, CA, USA].

### Quantitative real-time PCR analysis (Q-PCR)

Q-PCR was performed using Power SYBR Green PCR Master mix [Life Technologies (4367659)] with a CFX384 real-time qPCR system [Bio-Rad, Hercules, CA, USA] according to the manufacturer's instructions. The primer sequences are listed in Supplementary Table S3. Most of the primer sets were designed using Primer-Blast (NCBI) within a distance of 500 base pairs (bp) from the TTSs.

### Library construction for the SC3-seq for the SOLiD 5500XL system

Five nanogram of amplified and quality-checked cDNAs were added to the pre-amplification buffer [1 × ExTaq buffer [Takara Bio (RR006), Shiga, Japan], 0.2 mM of each dNTP [Takara Bio (RR006)], 0.01 μg/μl of the N-V3 (dT)_24_ primer (HPLC-purified, attachment of amine at the 5-prime end), 0.01 μg/μl of the V1(dT)_24_ primer (HPLC-purified) and 0.025 U/μl of ExTaqHS [Takara Bio (RR006)]], and were amplified by four cycles of PCR. The surplus primers and PCR byproducts such as primer dimers were removed by size selection through three rounds of purification using a 0.6 × volume of AMPureXP beads for each round [Beckman Coulter (A63881), Tokyo, Japan] according to the manufacturer's instructions. The purified cDNAs were diluted to 130 μl by DDW and fragmented by shearing with Covaris S2 or E210 (duty cycle: 20%; intensity: 5; cycles per burst: 200; duration: 50 s; an intensifier was installed in the case of E210) [Covaris, Woburn, MA, USA] and then end-polished in the End-polish buffer [1 × NEBnext End Repair Reaction buffer [NEB (B6052S), Ipswich, MA, USA], 0.01 U/μl of T4 DNA polymerase [NEB (M0203)] and 0.033 U/μl of T4 polynucleotide kinase [NEB (M0201)]] for 30 min at 20ºC. After incubation, a 0.8 × volume of the AMPureXP was immediately added, the solution was mixed for more than 20 min and then the supernatant was transferred to a 1.2 × volume of the AMPureXP reagent and the cDNAs were purified. Next, to provide the purified cDNAs with an Int-adaptor sequence, the cDNAs were incubated in 30 μl of the Internal adaptor extension buffer [1 × ExTaq Buffer, 0.23 mM of each dNTP, 0.67 μM of the IntV1 (dT)_24_ primer (HPLC-purified), 0.033 U/μl of ExTaqHS] using the following thermal cycler program: 95ºC for 3 min; 67ºC for 2 min; and 72ºC for 2 min. The reactions were terminated by chilling in an ice-block, and after the addition of 20 μl of the P1-adaptor ligation buffer [a mixture of 10 μl of 5 × NEBNext Quick Ligation Reaction Buffer [NEB (B6058S)], 0.6 μl of 5 μM of the P1-T adaptor [Life Technologies (4464411)] and 1 μl of T4 ligase [NEB (M0202M)]], the solution was incubated for 15 min at 20ºC and for 20 min at 72ºC. After two rounds of cDNA purification by adding a 1.2 × volume of AMPure XP, the cDNAs were added into the Final amplification buffer [1 × ExTaq buffer, 0.2 mM of each dNTP, 1 μM of the P1 primer, 1 μM of the BarT0XX_IntV1 primer (HPLC-purified), 0.025 U/μl of ExTaqHS] and amplified by PCR using the following thermal cycler program: 95ºC for 3 min; followed by nine cycles of 95ºC for 30 s, 67ºC for 1 min and 72ºC for 1 min; with a final extension of 72ºC for 3 min. Finally, the cDNA libraries were purified two times by using a 1.2 × volume of AMPureXP and dissolved in 20 μl of TE buffer. The quality and quantity of the constructed libraries were evaluated by LabChip GX or Bioanalyzer 2100, a Qubit dsDNA HS assay kit [Life Technologies (Q32851)] and a SOLiD Library TaqMan Quantitation kit [Life Technologies (4449639)]. The clonal amplification of the libraries on beads by emulsion PCR (emPCR) was performed using SOLiD™ EZ Bead™ System [Life Technologies (4449639)] at the E120 scale according to the manufacturer's instruction. The resulting bead libraries were loaded into flowchips and sequenced for 50 bp and 5-bp barcode plus Exact Call Chemistry (ECC) on an SOLiD 5500XL system [Life Technologies (4449639)].

### Mapping of the raw reads to the reference genome

All the reads were surveyed and the adaptor or the poly-A sequences were trimmed by cutadapt-1.3 (28). The trimmed reads with less than 30 bp were discarded. The adaptor and the poly-A sequences were observed in ∼1–20% and 5% of the total reads, respectively. Untrimmed and trimmed reads of 30 bp or longer were mapped onto the mouse genome mm10 and the ERCC spike-in RNA sequences with tophat-1.4.1/bowtie1.0.1 with the ‘—no-coverage-search’ option (29). Mapped reads on the genome and the ERCC were separated, and the reads on the genome were converted into the expression levels by cufflinks-2.2.0 using the ‘—compatible-hits-norm’, ‘—no-length-correction’ and ‘—library-type fr-secondstrand’ options and mm10 reference gene annotations with extended TTSs (30). We also set the cufflinks option ‘—max-mle-iterations’ to 50 000, because default iterations (5000) resulted in ‘FAILED’ when estimating the expression levels of some genes. For the reference gene annotations used in cufflinks, we extended the TTSs of the reference genes up to 10 kb downstream to correctly estimate the expression levels of genes whose transcripts are longer than the reference toward the 3 prime. To estimate the transcript copy number per cell, the ERCC spike-in RNA reads were normalized to reads per million-mapped reads (RPM) by total mapped reads on the genome used for gene expression analysis. Mapped reads were visualized using igv-2.3.34 (31). We confirmed that the conversion of the mapped reads into the expression levels by HTSeq-0.6.0 (32) gave similar results to that by cufflinks-2.2.0 using the described options (data not shown).

### Data analysis for the performance of the SC3-seq and the other published methodologies

Analysis was performed using R software version 3.0.2 and Excel (Microsoft). The expression data by the SC3-seq were analyzed using log_2_ (RPM+1) as the expression value, except in Figure 4 and Supplementary Figure S4A and B. In Figure 4, the RPM/FPKM values less than 0.01 were set as 0.01 for the calculation of correlation coefficients (13,33,34). In Supplementary Figure S4A and B, the RPM/FPKM values less than 0.1 were set as 0.1 for the calculation of correlation coefficients (13).

**Figure 1.**
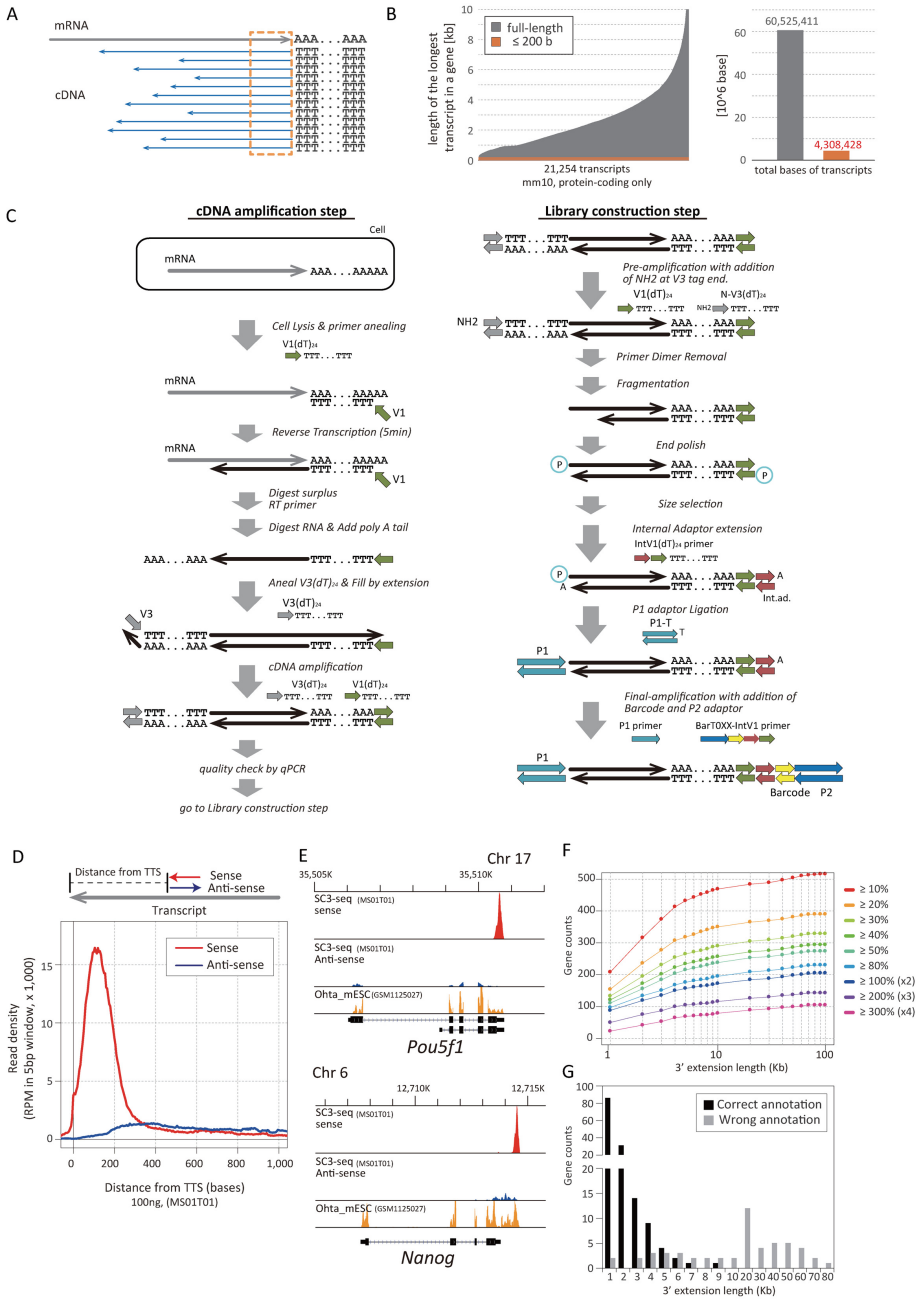
Establishment of the SC3-seq. (**A**) Concept of the SC3-seq. Synthesis and amplification of full-length cDNAs by reverse transcription (RT) and PCR, respectively, are not efficient processes due to a number of potential limitations in RT and PCR. The SC3-seq targets only the 3-prime ends of the transcripts (orange dotted square), which are more faithfully/quantitatively reverse-transcribed and amplified by RT and PCR, respectively, which in turn will lead to a more quantitative outcome of the sequence reads for all genes. (**B**) 21 254 protein-coding genes annotated in the Mouse mm10 database are aligned in the order of the length of their longest transcripts. While the sum of all the longest transcript lengths is around 60 Mbp, that of the 200 bp from the 3-prime ends of all transcripts is only around 4 Mbp, allowing sequence saturation at much smaller sequence depth. (**C**) Scheme for the SC3-seq, which consists of (left) cDNA synthesis and amplification, and (right) library construction. In the first step (left), V1 (green) and V3 (gray) tags are added at the 3- and 5-prime ends of cDNAs, respectively, as reported previously (7,8). In the second step (right), tags for the SOLiD sequencing (P1 tag, light blue; P2 tag, dark blue; Internal adaptor, red; index for multiplexing, yellow) were added sequentially as depicted. The 3-prime ends of the cDNAs are captured in the Internal adaptor extension step. See the Materials and Methods section and Supplementary Figure S1A for more details. (**D**) The averaged SC3-seq track [read density (RPM, ×1000 reads) plotted against the read position from the annotated TTSs] of 100 ng (10 000-cell level) of total RNAs from mESCs. The red line represents the track of reads mapped on the sense strands; the blue line shows the track of reads mapped on the anti-sense strands. (**E**) The SC3-seq reads of the *Pou5f1* and *Nanog* loci. The red peaks indicate the reads mapped on the sense strands; the blue peaks show the reads mapped on the anti-sense strands. The orange peaks are the reads mapped by Ohta *et al*. (33). (**F**) Gene counts showing the increase in the number of reads as indicated by the color code plotted against the extent of the definition of the 3-prime end extension. (**G**) Gene counts for correct (black bars) or wrong (gray gars) annotations by the extension of the definition of the TTSs by the indicated length. The 205 genes that exhibited gene counts of ≧2-fold (×2, ×3, ×4 in Figure 1F) by extending the definition of the TTSs by 10 Kb were visually inspected for collect/wrong annotations in comparison to the published RNA-seq data (33).

**Figure 2.**
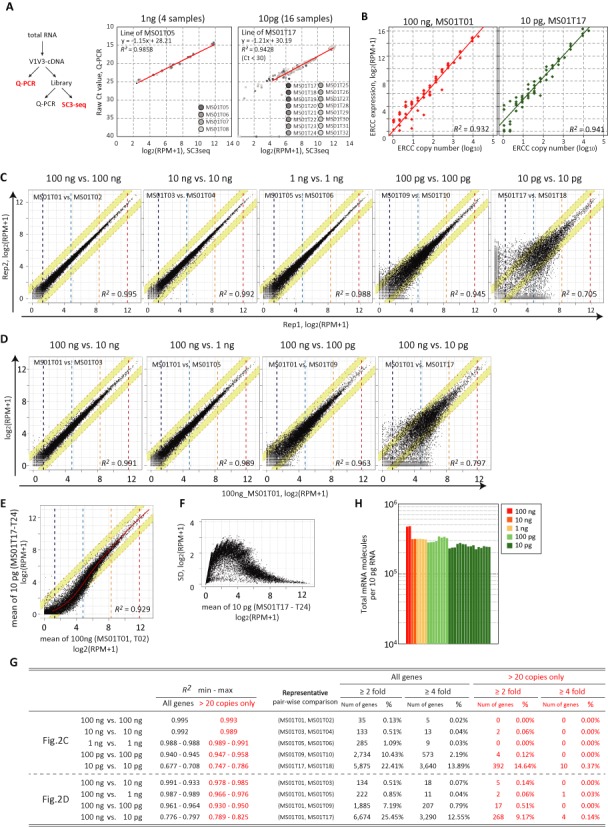
Quantitative performance of the SC3-seq. (**A**) Comparison of the expression levels of the genes listed in Supplementary Table S3 in amplified cDNAs [from 1 ng (left, four replicates) and 10 pg (right, 16 replicates) total RNAs, prior to the SC3-seq library construction] estimated by Q-PCR (raw Ct value) with those estimated by the SC3-seq [log_2_ (RPM+1)]. Regression lines of representative samples are shown (MS01T05 and MS01T17 for 1 ng and 10 pg, respectively, of total RNAs). (**B**) Representative examples for correlations between the quantities of the ERCC RNAs spiked in a dilution series of mESC total RNAs (MS01T01 and MS01T17 for 100 ng and 10 pg of total RNAs, respectively) and the estimated levels of the spike-in RNAs by the SC3-seq [log_2_ (RPM+1)]. The SC3-seq data for the ERCC spike-in RNAs with more than 10 copies per 10 pg are used for the regression line. See Supplementary Figure S3A for correlations in all samples. (**C**) Scatter-plot comparison of the SC3-seq [log_2_ (RPM+1)] between two independently amplified replicates from 100 ng, 10 ng, 1 ng, 100 pg and 10 pg of mESC total RNAs. The white and yellow areas indicate expression-level ranges within 2-fold and 4-fold differences between the replicates, respectively (also in (**D**) and (**E**)). Copy numbers per 10 pg of total RNAs estimated by the SC3-seq reads of the ERCC spike-in RNAs in 100 ng of RNAs are indicated by dashed lines (red: 1000 copies; orange: 100 copies; pale blue: 10 copies; blue: 1 copy) (also in D–**F**). (D) Scatter-plot comparison of the SC3-seq [log_2_ (RPM+1)] between a replicate from 100 ng of mESC total RNAs and a replicate from 10 ng, 1 ng, 100 pg and 10 pg of mESC total RNAs. (E) Scatter-plot comparison of the averaged SC3-seq data [log_2_ (RPM+1)] of 100 ng of mESC total RNAs (two replicates) with those of 10 pg of RNAs (eight replicates). (F) Standard deviations (SDs) of gene-expression levels plotted against mean gene-expression levels by the SC3-seq in the indicated eight 10-pg RNA samples. (**G**) Correlations in indicated comparisons [minimum (min) and maximum (max) correlation coefficients (*R*^2^) are shown] and the percentages of genes within 2- and 4-fold expression differences in the indicated representative comparisons (all expression ranges and genes expressed more than 20 copies per 10 pg of RNAs). (**H**) Total mRNA molecule numbers per 10-pg RNA estimated from the copy numbers of the ERCC spike-in RNAs in 100 ng, 10 ng, 1 ng, 100 pg and 10 pg of mESC total RNAs.

**Figure 3.**
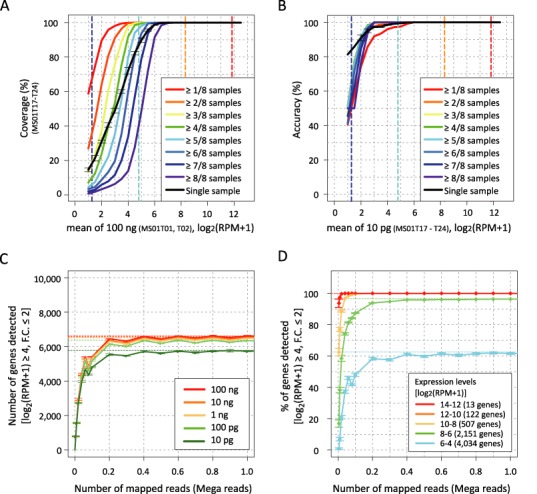
Coverage and accuracy of the SC3-seq. (**A**) Coverage of the SC3-seq from 10 pg of total RNAs as a function of the expression level [log_2_ (RPM+1)] in 100 ng of total RNAs. The black squares represent the means of coverage in single-sample analysis, with bars representing SDs. The results of multiple-sample analyses under the definitions of detection where transcripts are detected in ≧1–8 of the eight amplified samples are represented by squares with the indicated color codes. Copy numbers per 10 pg of total RNAs estimated by the SC3-seq reads of the ERCC spike-in RNAs in 100 ng of RNAs are indicated by dashed lines (red: 1000 copies; orange: 100 copies; pale blue: 10 copies; blue: 1 copy). (**B**) Accuracy of the SC3-seq from 10 pg of total RNAs as a function of the expression level [log_2_ (RPM+1)]. The representation code is the same as in (A). (**C**) The plot of the number of genes detected [log_2_ (RPM+1) ≧ 4, fold changes of gene expression levels ≦2 in comparison to those determined by the full sequence reads (Supplementary Table S1)] by the SC3-seq from 100 ng, 10 ng, 1 ng, 100 pg and 10 pg of mESC total RNAs (color code indicated) as a function of the sequence reads. (**D**) The plot of the percentage of the detection [fold changes of gene expression levels ≦2 in comparison to those determined by the full sequence reads (Supplementary Table S1)] by the SC3-seq from 10 pg of total RNAs as a function of the sequence reads, categorized by expression level ranges in 100 ng of total RNAs (color code indicated).

**Figure 4.**
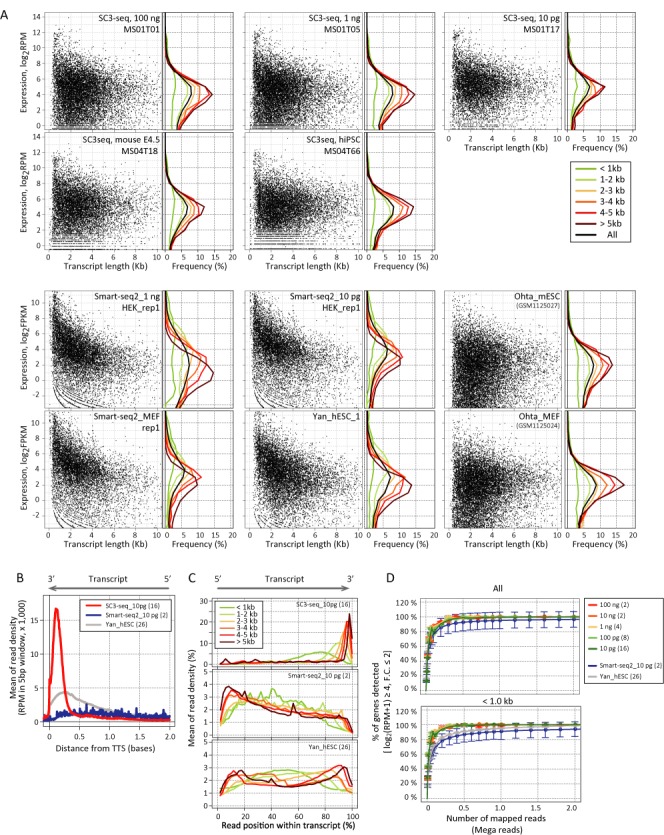
Comparison of the performance of the SC3-seq with that of other single-cell RNA-seq methods. (**A**) Scatter-plot analysis of the correlation between the expression level and the transcript length, detected by the SC3-seq [dilution samples from 100 ng (replicate 1, MS01T01), 10 ng (replicate 1, MS01T05) and 10 pg (replicate 1, MS01T17) of ESC total RNAs, and a single mouse embryonic cell (MS04T18) and a single human iPSC (MS04T66)], the Smart-seq2 [dilution samples from 1 ng (Smart-seq2_1ng, HEK_rep1) and 10 pg (Smart-seq2_10 pg, HEK_rep1) of HEK293 total RNAs, and a single mouse embryonic fibroblast (Smart-seq2_MEF, rep 1)] (13), the single-cell RNA-seq by Yan *et al*. [a single human ESC (Yan_hESC_1)] (34), and a full-length RNA-seq [Ohta_mESC and Ohta_MEF (33)]. The expression levels by the SC3-seq are shown as log_2_ RPM, whereas those by the other methods are shown as log_2_ FPKM. The histogram on the right of each scatter plot indicates the distribution of gene-expression levels of transcripts with different length ranges (color code indicated). (**B**) Distributions of the mapped reads around the 3-prime ends of the transcripts by the three single-cell RNA-seq methodologies. (**C**) Distributions of the mapped reads within the whole transcripts of different length ranges (color code indicated) by the three single-cell RNA-seq methodologies. (**D**) Analysis for saturation of detection [gene-expression levels ≧ the top 6555th and 6217th genes for mice and humans, respectively (1/4 of all the annotated transcripts for mice and humans), ∼log_2_ RPM ≧ 3.69 ± 0.05 (SC3-seq), ∼log_2_ FPKM ≧ 2.21 ± 1.28 (Yan *et al*.) and ∼log_2_ FPKM ≧ 2.92 ± 0.27 (Picelli *et al*.), fold changes of gene expression levels ≦ 2 in comparison to those determined by the full sequence reads [Supplementary Table S1 or (13,34)] for all (top) and short (<1 kb, 913 and 832 genes for mice and humans, respectively) transcripts by the three single-cell RNA-seq methodologies. See Supplementary Figure S4C for transcripts <750 bp and <500 bp.

The estimation of the optimum definition of the 3-prime ends of the transcripts was performed by modifying the reference gene annotation gff3 file. We calculated all gene expression values when the definition of the 3-prime ends of the transcripts was extended in 1-kb increments up to 10 kb, then in 10-kb increments up to 100 kb. Genes whose expression levels were increased by 10, 20, 30, 40, 50, 80, 100, 200 and 300% were counted.

The estimation of the approximate copy numbers per 10 pg total RNAs was performed by drawing a single linear regression line for all log_2_ (RPM+1) values of the ERCC spike-in RNAs (excluding the ERCC spike-in RNAs whose copy numbers are lower than 100 copy per 10-pg RNAs and the two outliers ERCC-00116 and ERCC-00004) in all the 32 amplified samples as a function of their copy numbers. The approximate log_2_ (RPM+1) values for 1000 copies, 100 copies, 10 copies and one copy per 10 pg total RNA were 11.78, 8.10, 4.43 and 0.76, respectively.

The coverage is defined by the number of genes detected in samples amplified from 10 pg of total RNAs [log_2_ (RPM+1) ≧1] as a percentage of that in samples amplified from 100 ng of total RNAs [log_2_ (RPM+1) ≧1] for different expression-level ranges. Accuracy is defined based on the number of genes detected in samples amplified from 100 ng of total RNAs [log_2_ (RPM+1) ≧1] as a percentage of that in samples amplified from 10 pg of total RNAs [log_2_ (RPM+1) ≧1]. The truly expressed genes were defined as those that were detected [log_2_ (RPM+1) ≧1] in both samples prepared by SC3-seq from 100 ng of RNAs. Multiple sample analyses (eight samples) for the coverage and the accuracy were performed by calculating the coverage and accuracy under definitions of detection where ≧1 to ≧8 of the eight amplified samples exhibited reads.

For analysis of the saturation of the detection of gene expression, conversion of the mapped reads to gene-expression levels by cufflinks was repeated with the number of mapped reads reduced to as low as 10 000. The numbers of genes detected at significant expression levels [log_2_ (RPM+1) > 4] were counted under different numbers of total mapped reads. When comparing the data by the SC3-seq with the published data by the other methodologies, we used log_2_ RPM as an expression value for the SC3-seq and included the top 6555th and 6217th genes for mice and humans, respectively, (1/4 of all the annotated transcripts for mice and humans) for the analysis.

### Data analysis of the SC3-seq results for the single cells in the mouse E4.5 blastocysts and hiPSCs

Analysis was performed using R software version 3.1.1 with the gplots and the qvalue packages, and EXCEL (Microsoft). All the analyses of expression data were performed using log_2_ (RPM+1) values. Genes whose log_2_ (RPM+1) value was less than 4 (less than ∼20 copies/cell) in all samples were excluded from the analysis. Unsupervised hierarchical clustering (UHC) was performed using the hclust function with Euclidean distances and Ward distance functions (ward.D2). The principal component analysis (PCA) was performed using the prcomp function without scaling. To identify differentially expressed genes (DEGs) among multi-groups, the oneway_analysis of variance (ANOVA) and the qvalue function were used for the calculation of the *P* value and false discovery ratio (FDR), respectively. The DEGs were defined as those exhibited a more than 4-fold change between the samples (FDR < 0.01), and the mean of the expression level of the group was ≧ log_2_ (RPM+1) = 4. The gene ontology (GO) analysis was performed using the DAVID web tool (35).

### Immunofluorescence analysis of mouse E4.5 embryos

For whole-mount immunofluorescence (IF) analysis, isolated embryos were fixed in 4% paraformaldehyde in PBS for 20 min at room temperature, washed in 2% bovine serum albumin (BSA)/PBS and incubated in the permeabilization solution (0.5% Triton X/1.0% BSA/PBS) for 20 min at room temperature. After washing twice in 2% BSA/PBS, embryos were incubated with primary antibodies in 2% BSA/PBS overnight at 4%, washed three times with 2% BSA/PBS, incubated with secondary antibodies and 4,6-diamidino-2-phenylindole (DAPI) in 2% BSA/PBS for 1 h at room temperature, washed three times with 2% BSA/PBS and mounted in VECTASHIELD Mounting Medium [Vector Laboratories, (H-1000)]. The primary antibodies used were as follows: anti-mouse NANOG [rat monoclonal; eBioscience, (eBio14-5761)], anti-mouse POU5F1 [mouse monoclonal; Santa Cruz, (sc-5279)], anti-mouse GATA4 [goat polyclonal; Santa Cruz, (sc-1237)], anti-mouse CDX2 [rabbit monoclonal, clone EPR2764Y; Abcam, (ab76541)]. The secondary antibodies used were as follows: Alexa Fluor 488 anti-rat IgG [Life Technologies (A21208)], Alexa Fluor 555 anti-rabbit [Life Technologies (A31572)], Alexa Fluor 568 anti-mouse IgG [Life Technologies (A10037)] and Alexa Fluor 647 anti-goat IgG [Life Technologies (A21447)] (all donkey polyclonal). Image data were obtained and processed by a confocal microscope [Olympus, (FV1000)].

## RESULTS

### Design and establishment of the SC3-seq

We have previously reported a method for the amplification of single-cell cDNAs enriched in the 3-prime ends (687 ± 279 bp) for high-density oligonucleotide microarray analysis (7,8). The method has been successfully used for the analysis of diverse biological processes, including the identification of cell-type heterogeneity/specification in mouse blastocysts (7,36), the identification of transcriptome dynamics during the specification and development of primordial germ cells (37) and the identification of neuronal subtypes during cerebellar cortex development (38). The method has been shown to be powerful not only for the transcriptome analysis of single cells but also for cells in the range of several hundred to thousands (22,39). The method has subsequently been modified so that longer cDNAs including full-length cDNAs are synthesized and analyzed by RNA-seq (9–11). However, considering the inefficiency of full-length cDNA synthesis and susceptibility to amplification bias of longer cDNAs, amplification and sequencing of the 3-prime ends of cDNAs should provide more precise assessment of gene-expression levels (Figure 1A). Furthermore, sequencing of only the 3-prime ends theoretically requires a much smaller sequence depth for saturation (Figure 1B), making the analysis more cost-effective.

We therefore set out to design and establish a methodology that amplifies and sequences the 3-prime ends of cDNAs synthesized from single cells, which we designate the SC3-seq (Single-cell mRNA 3-prime end sequencing) method (Figure 1C). For the amplification of cDNAs, we adopt essentially the same methodologies we reported previously, which amplify cDNAs from single cells/single-cell level RNAs in a highly representative manner (7,8) (Figure 1C). The first-strand cDNAs are synthesized by the V1 (dT)_24_ primer, the excess V1 (dT)_24_ primer and the annealed mRNAs are digested by Exonuclease I and RNaseH, respectively, the poly (dA) tail is added at the 3-prime ends of the first-strand cDNAs, the second-strand cDNAs are synthesized by the V3 (dT)_24_ primer and the resultant cDNAs are amplified by the V1 (dT)_24_ and V3(dT)_24_ primer pair by a number of PCR cycles depending on the amount of the starting materials (20 cycles for single cells or 10 pg of total RNA). For the construction of the library for sequencing by the SOLiD sequencer, we design a procedure that enriches the very 3-prime ends of the cDNAs bearing the V1(dT)_24_ primer (Figure 1C and Supplementary Figure S1A). The amplified cDNAs are tagged by the NH2-V3(dT)_24_ primer with a few PCR cycles, the primer dimers are removed by three rounds of purification by AMPureXP and the tagged cDNAs are fragmented by sonication, end-polished and size-fractionated by sequential purification by AMPure XP. The resultant cDNAs of ∼200–250 bp in size are denatured, annealed by the IntV1(dT)_24_ primer for the capture of the 3-prime ends with the extension of the internal adaptor extension sequence, ligated and sequence-extended with the P1 adaptor and processed for final amplification by the P1 primer and the BarT0XX-IntV1 primer bearing 96 distinct barcodes (a larger number of barcodes can be provided if desired). The final amplified products are sequenced from the P1-adaptor end, resulting in the mapping of the very 3-prime ends of the mRNAs on the genomic loci. Since the SC3-seq provides sequence reads only at the very 3-prime ends of mRNAs, the absolute read counts become proportional to the expression levels of mRNAs irrespective of their entire length, allowing simple and more accurate quantification of the gene-expression levels. Normalization by sequence reads per million-mapped reads should thus provide an accurate quantification.

To examine the performance of the SC3-seq, we made a dilution series of total RNAs [100 ng (10 000-cells level), two replicates; 10 ng (1000-cells level), two replicates; 1 ng (100-cells level), four replicates; 100 pg (10-cells level), eight replicates; and 10 pg (single-cell level), 16 replicates] isolated from mESCs, and, using these RNAs together with a set of external spike-in RNA controls developed by the External RNA Controls Consortium (ERCC), amplified (7, 11, 14, 17 and 20 initial PCR cycles for 100 ng, 10 ng, 1 ng, 100 pg and 10 pg total RNAs, respectively) and sequenced them by the SC3-seq. We confirmed by quantitative (Q)-PCR that the ERCC spike-in RNAs were amplified highly proportionally to their spiked-in copy numbers during the initial cDNA amplification (Supplementary Figure S1B) and that the representation of a number of genes expressed in ESCs (Supplementary Table S3) in the final cDNA library just prior to sequencing is essentially identical to that in the initially amplified cDNAs (Supplementary Figure S1C), demonstrating the preservation of the representation of the gene contents during both the cDNA amplification and library construction processes for the SC3-seq.

Figure 1D shows the averaged sequence read distribution of one of the amplified products of the 100-ng RNAs by the SC3-seq (∼40–50% mapping efficiency; Supplementary Table S1). In good agreement with the design of SC3-seq, the mapped reads were highly enriched at the very 3-prime ends (∼150 bp upstream from the TTSs) of nearly all mapped RefSeq genes, including non-coding RNAs (Supplementary Figure S2). We found minor enrichment of the mapped reads on the anti-sense strands of exons as well, which would in part represent amplification products in which the V1 (dT)_24_ primer was mis-annealed to the 5-prime ends of cDNAs for amplification (Figure 1C). For example, the SC3-seq track around the *Pou5f1* locus exhibited a single clear peak corresponding to the very 3-prime end of the sense strand of *Pou5f1*, with several minor peaks on the anti-sense strand of the exons (Figure 1E). We also found that, for a fraction of genes, the peaks of the SC3-seq reads were observed downstream of the 3-prime ends of annotated RefSeq transcripts, suggesting that these loci correspond to the ‘bona fide’ 3-prime ends of the upstream transcripts (Figure 1D and E). For example, for the *Nanog* locus, the SC3-seq peaks were observed ∼ 1 kb downstream of the annotated 3-prime end of *Nanog* (Figure 1E). Indeed, examination of the published RNA-seq data for a large amount of RNAs of mESCs with massive sequence depth (33) identifies the same peak contiguous to the peaks of the upstream exons of *Nanog* (Figure 1E), indicating that the 3-prime end peaks detected by the SC3-seq belong to *Nanog* transcripts. This finding led us to re-define the 3-prime ends of a fraction of the RefSeq transcripts. We examined the correlation between the extent of *in silico* extension of the TTSs of the RefSeq transcripts and the gene numbers that show an increase in mapped reads by the SC3-seq. As shown in Figure 1F, the number of genes that exhibited the increase of mapped reads was increased by the extension of the definition of the TTSs from the annotated TTSs, and ∼450 genes exhibited the increase of their mapped reads by the extension of the definition of the TTSs by 10 kb. We inspected the 205 genes that exhibited gene counts of ≧ 2-fold (×2, ×3, ×4 in Figure 1F) by extending the definition of the TTSs by 10 kb in comparison to the published RNA-seq data (33) and found that in mESCs, while the extension by 10 kb indeed recovered primarily correct gene counts, the extension by more than 10 kb resulted in primarily incorrect annotations (Figure 1G). We therefore decided to define the peaks detected within 10 kb downstream from the annotated TTSs as indicating the expression of their immediate up-stream genes and to analyze the SC3-seq data accordingly.

### Evaluation of the quantitative performance of the SC3-seq

#### Reproducibility of the SC3-seq

To evaluate the quantitative performance of the SC3-seq, we first analyzed the correlation between the threshold-cycle (Ct) values by quantitative (Q)-PCR of a number of genes in amplified cDNAs from 1 ng (100-cell level, four replicates) and 10 pg (1-cell level, 16 replicates) of total RNAs and the SC3-seq reads [log_2_ (RPM+1)] of the same set of genes in the library prepared from the same cDNAs. As shown in Figure 2A, remarkably, the Ct values and the SC3-seq reads exhibited superb correlations for both cDNAs (*R*^2^ = 0.9858 and 0.9428 for representative cDNAs from 1-ng and 10-pg RNAs, respectively). Consistently, the SC3-seq reads [log_2_ (RPM+1)] of the ERCC spike-in RNAs in amplified libraries of all dilution series correlated highly proportionally to their original copy numbers (Figure 2B and Supplementary Figure S3A; see Supplementary Table S2 for the copy numbers of ERCC spike-in RNAs provided per 10 pg total RNAs in each dilution), although the spike RNAs that provided less than ∼30 copies in 10-pg RNAs exhibited somewhat less efficient amplification. This makes it possible to estimate the copy number per 10-pg RNAs of a gene from its SC3-seq read number.

The scatter-plot analyses indicated that independent samples amplified from 100 ng (10 000 cell-level, two replicates), 10 ng (1000 cell-level, two replicates) and 1 ng (100 cell-level, four replicates) RNAs exhibited excellent correlations (*R*^2^ = 0.995, 0.992, 0.988, respectively), with nearly all the genes plotted within the 2-fold difference lines (Figure 2C and G and Supplementary Figure S3B and C). The samples amplified from 100 pg (10-cell level, eight replicates) RNAs also showed very good correlations (min *R*^2^–max *R*^2^ = 0.940–0.945), with 89.6 and 97.8% of genes in a representative pair-wise comparison (all expression ranges) plotted within the 2- and 4-fold difference lines, respectively (Figure 2C and G and Supplementary Figure S3B and C). The samples amplified from 10 pg (1-cell level, 16 replicates) RNAs exhibited good correlations (min *R*^2^–max *R*^2^ = 0.677–0.708), with 77.6 and 86.1% of genes in a representative pair-wise comparison (all expression ranges) plotted within the 2- and 4-fold difference lines, respectively (Figure 2C and G and Supplementary Figure S3B and C). We noted that independently amplified samples showed better correlations for genes that are expressed more than ∼20 copies per 10 pg of RNAs, and particularly for those amplified from 10 pg of RNAs, with min *R*^2^–max *R*^2^ = 0.747–0.786, and with 85.4 and 99.6% of genes in a representative pair-wise comparison plotted within the 2- and 4-fold difference lines, respectively (Figure 2C and G and Supplementary Figure S3B and C).

When compared to the sequence profiles of samples amplified from 100 ng (10 000 cell-level) of RNAs, samples amplified from 10 ng (1000 cell-level), 1 ng (100 cell-level) and 100 pg (10-cell level) of RNAs exhibited very good correlations (min *R*^2^–max *R*^2^ = 0.991–0.993, 0.987–0.989, 0.961–0.964, respectively), and those amplified from 10 pg (1-cell level) RNAs showed good correlations (min *R*^2^–max *R*^2^ = 0.776–0.797), with 75 and 87% of genes in a representative pair-wise comparison (all expression ranges) plotted within the 2- and 4-fold difference lines, respectively (Figure 2D and G and Supplementary Figure S3B and C). For genes that are expressed more than ∼20 copies per 10 pg of RNAs, samples amplified from 10 pg (1-cell level) RNAs showed better correlations (min *R*^2^–max *R*^2^ = 0.789–0.825), with 90.8 and 99.9% of genes in a representative pair-wise comparison plotted within the 2- and 4-fold difference lines, respectively (Figure 2D and G and Supplementary Figure S3B and C).

To evaluate the performance of the SC3-seq for 1-cell level RNAs further, we compared the log-averaged expression levels in two samples amplified from 100 ng of RNAs with those in eight samples amplified from 10 pg of RNAs. The scatter-plot analysis showed that the averaged samples exhibited a better correlation (*R*^2^ = 0.929), with 79.8 and 97% of genes (all expression ranges) plotted within the 2- and 4-fold difference lines, respectively (Figure 2E). For genes that are expressed more than ∼20 copies per 10 pg of RNAs, they showed a good correlation (*R*^2^ = 0.922), with 98.6 and > 99.9% of genes plotted within the 2- and 4-fold difference lines, respectively, and exhibited relatively small standard deviations (SDs) of expression levels (Figure 2E and F). Collectively, these findings demonstrate the highly quantitative performance of the SC3-seq for RNAs ranging from 100 ng (10 000-cell level) to 10 pg (1-cell level). Based on these results, we estimated the number of mRNA molecules present in 10 pg (1-cell level) of mESC total RNAs as ∼300 000 (Figure 2H), a value in good agreement with previous findings (1,16).

#### Coverage and accuracy by the SC3-seq

To examine the performance of the SC3-seq further, we next evaluated the coverage [the number of genes detected in 10-pg RNAs (log_2_ (RPM+1) ≧1)/the number of genes detected in 100-ng RNAs (log_2_ (RPM+1) ≧1)] and accuracy [the number of genes detected in 10-pg RNAs (log_2_ (RPM+1) ≧1) that are detected in 100-ng RNAs (log_2_ (RPM+1) ≧1)] of the SC3-seq from 10-pg RNAs by using the data from eight replicates. The truly expressed genes were defined as those that were detected [log_2_ (RPM+1) ≧1] in both samples prepared by SC3-seq from 100 ng of RNAs. Coverage of the single amplified samples as a function of the expression level was plotted (black squares in Figure 3A). As expected from the previous reports (7) and the data shown above (Figure 2), coverage was dependent on the expression level, but a vast majority of the truly expressed genes (cumulative percentage, 94.1%) that are expressed more than 10 copies per 10 pg were successfully detected (Figure 3A). The accuracy of the single amplified samples was plotted similarly, and we found that 99.7% (cumulative percentage) of the genes detected were truly expressed in the expression level range of more than 10 copies per 10-pg RNAs (Figure 3B). When we performed multiple sample analyses (eight samples were analyzed), coverage was improved under the definitions of detection where ≧1 to ≧5 of the eight amplified samples exhibited reads (≧10 copies per 10-pg RNAs), whereas accuracy was essentially nearly 100% under all detection definitions (Figure 3A and B). These findings indicate that a single sample prepared by SC3-seq from single-cell level RNAs exhibits excellent coverage and accuracy, and multiple sample analyses further improve both the coverage and accuracy.

#### Sequence reads required for the SC3-seq

One of the proposed advantages of the SC3-seq is that it allows precise quantification of gene expression with a relatively small depth of sequence reads. Next, therefore, we went on to examine the number of reads that would be sufficient to accurately determine gene-expression levels within from 100-ng (10 000-cell level) to 10-pg (1-cell level) RNAs. We plotted the number of genes with log_2_ (RPM+1) ≧ 4 (≧ 5 copies per 10-pg RNAs) that were detected accurately against the read numbers [fold changes of gene expression levels ≦2 in comparison to those determined by the full sequence reads (Supplementary Table S1)]. As shown in Figure 3C, the number of genes detected with [log_2_ (RPM+1) ≧ 4, fold changes of gene expression levels ≦ 2] in RNAs from 100 ng (10 000-cell level) to 10 pg (1-cell level) by SC3-seq became saturated at around 0.2 mega (M) mapped reads and that the detected gene number was essentially identical in RNAs from 100 ng (10 000-cell level) to 100 pg (10-cell level) (∼7000) and was smaller in 10-pg RNAs (1-cell level) (∼6000). We then plotted the percentage of genes accurately detected by the SC3-seq from 10 pg of total RNAs among genes detected from 100 ng of total RNAs against the sequence reads, categorized by expression-level ranges [fold changes of gene expression levels ≦2 in comparison to those determined by the full sequence reads (Supplementary Table S1)] (Figure 3D). This analysis also revealed that nearly all the genes with log_2_ (RPM+1) ≧ 6 (≧ 20 copies per 10-pg RNAs) and ∼60% of genes with log_2_ (RPM+1) ≧ 4 (≧ 5 copies per 10-pg RNAs) were detected in saturation by the SC3-seq from 10 pg of total RNAs by around 0.2-M mapped reads (Figure 3D). We therefore conclude that to identify genes expressed at a substantial level [log_2_ (RPM+1) ≧ 4, ≧ 5 copies per 10-pg RNAs] by SC3-seq, it should be sufficient to perform 0.2-M mapped reads per sample, a substantially small number that allows a highly parallel sequencing of numerous samples.

### Comparison of the performance of SC3-seq with that of other typical methods

We next compared the quantitative performance of the SC3-seq with that of other typical methods for single-cell RNA seq (9,10,13,34).

First, we compared the reproducibility of the transcript measurements in diluted RNAs by the SC3-seq with that by the Smart-seq2 (13) [the manuscripts by (9,10,34) did not examine the reproducibility of their method using a dilution series of RNAs]. As shown in Supplementary Figure S4A and B, the maximum and minimum correlation coefficients (*R*^2^) of the transcript measurements in 10 pg of RNAs by the SC3-seq among 16 replicates were 0.689 and 0.659, respectively, whereas the correlation coefficient (*R*^2^) of the two replicates [only two replicates of 10 pg of RNAs were analyzed by (13)] by the Smart-seq2 was 0.613. Indeed, in all starting amounts of RNAs/in all pairs of comparisons, the maximum and minimum correlation coefficients (*R*^2^) of the transcript measurements by the SC3-seq were higher than those by the Smart-seq2 (Supplementary Figure S4A and B). These data strongly suggest that the SC3-seq shows superior reproducibility to the Smart-seq2.

Second, we examined the relationship between the estimated expression level and the transcript length in samples prepared by the SC3-seq and the other methods, and as a control, in the published RNA-seq data prepared from a large quantity of RNAs of mESCs and mouse embryonic fibroblasts (MEFs) with massive sequence depth (33). As shown in Figure 4A (panels at bottom right), the control samples exhibited a highly diverse distribution of expression-level ranges irrespective of the transcript lengths and cell types (Ohta_mESCs and Ohta_MEFs), and the average expression levels were similar among all transcript lengths [the modes of expression levels are around log_2_ FPKM (fragment per kilobase per million mapped reads) = 2]. Remarkably, the distribution of the expression-level ranges as a function of the transcript length detected by the SC3-seq for 100 ng, 1 ng and 10 pg of ESC RNAs as well as for single embryonic or cultured cells (see below) was very similar to that by the control RNA-seq (Figure 4A, panels in the upper rows): the genes show relatively constant expression levels irrespective of their transcript length, i.e. the modes of the expression levels are similar (around log_2_ RPM = 4) throughout all the transcript lengths. This finding indicates that the SC3-seq correctly reflects the representation of gene expression across all transcript-length ranges from a single-cell level starting material.

In contrast, both the results provided by Picelli *et al*. for 1 ng and 10 pg of RNAs from HEK293 cells and for single MEFs [Smart-seq2, (13)] and those reported by Yan *et al*. for single human (h) ESCs [a method that modified and adapted our amplification procedure for full-length RNA-seq, (9,10,34)] revealed a similar trend, which is markedly different from that by the SC3-seq and the control RNA-seq: the modes of expression levels vary depending on the transcript length (e.g. the modes of expression levels of longer genes were lower and those of shorter genes were higher) (Figure 4A, panels at bottom, two left columns), possibly due to the low efficiency of the synthesis/amplification for longer transcripts, which altered the representation of the original gene expression.

We next examined the differences of the read positions across the transcripts among the SC3-seq and the other single-cell RNA seq methods. As shown in Figure 4B and C, the SC3-seq exhibited a clear sharp peak exclusively at the very 3-prime ends of the transcripts across all the transcript-length ranges, as described in part above (Figure 1). Conversely, the other two methodologies, which should theoretically generate reads across the transcript length in a uniform manner, unexpectedly resulted in reads with somewhat non-uniform distribution along the transcripts and with a skew toward the 3-prime (Yan *et al*.) or 5-prime (Picelli *et al*.) ends, especially for longer transcripts (Figure 4C).

In accordance with these findings, we found that whereas the SC3-seq requires ∼0.2 M of mapped reads for saturation of the correct detection of short (<1 kb) transcripts [gene-expression levels ≧the top 6555th gene (the number corresponding to 1/4 of all the annotated transcripts in mice), ∼log_2_ RPM ≧ 3.69 ± 0.05, fold changes of gene expression levels ≦ 2 in comparison to those determined by the full sequence reads (Supplementary Table S1)], the other two methods require more than ∼2 M of mapped reads for saturation of the correct detection of such transcripts [<1 kb, gene-expression levels ≧the top 6555th and 6217th gene (the number corresponding to 1/4 of all the annotated transcripts in mice and humans, respectively), ∼log_2_ FPKM ≧ 2.92 ± 0.27 (13) or 2.21 ± 0.05 (34), fold changes of gene expression levels ≦ 2 in comparison to those determined by the full sequence reads (13,34)] (Figure 4D and Supplementary Figure S4C). Taken together, these results demonstrate that SC3-seq is a more quantitative and efficient technique for single-cell transcriptome analysis than the other two typical methods.

### Application of the SC3-seq to developmental/stem cell biology

#### Identification of genes that distinguish cell types in peri-implantation mouse blastocysts

The peri-implantation mouse blastocysts at embryonic day (E) 4.5 consist of at least three distinct cell types: the epiblasts, the primitive endoderm (PE) and the trophectoderm (TE) (40). The former two cell types make up the ICM. Based on the anatomical location, the TE can be classified into two types: the polar (p) TE, which directly contacts the ICM and subsequently forms extra-embryonic ectoderm (ExE) and ectoplacental cones, and the mural (m) TE, which is located at the ab-embryonic part of the blastocysts and later forms primary trophoblast giant cells (41,42). There has been no report exploring whether global gene-expression differences exist between pTE and mTE at E4.5. To demonstrate the capacity of the SC3-seq to address biologically relevant questions, we first went on to determine whether the SC3-seq successfully discriminates cell-type differences in the blastocysts.

**Figure 5.**
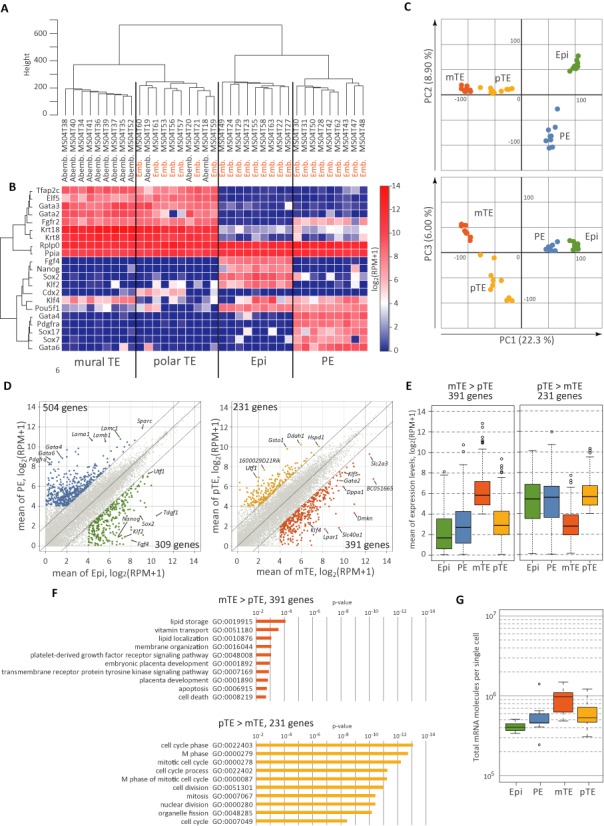
Analysis of E4.5 blastocysts by the SC3-seq. (**A, B**) Unsupervised hierarchical clustering (UHC) with all expressed genes [log_2_ (RPM+1) ≧ 4 in at least one sample, 12 010 genes] (A) and heat map representation of the expression levels of several marker genes for the epiblast, the primitive endoderm (PE) and the trophectoderm (TE) (B). The locations of the isolated cells (embryonic or ab-embryonic) are indicated between the clustering tree and the heat map. The annotated cell types [epiblast, PE, polar (p) TE and mural (m) TE] are defined based on the clustering, the locations of the isolated cells and known marker gene expression. (**C**) Principal component analysis (PCA) of all cells by all expressed genes. The cells [colored according to the clustering groups in (A)] are plotted in two-dimensional space defined by PC1 and PC2 (top) or by PC1 and PC3 (bottom). (**D**) Scatter-plot analysis of the averaged gene-expression differences between the epiblast (nine samples) and the PE (nine samples) (left), and between the mTE (nine samples) and the pTE (10 samples) (right). The differentially expressed genes are defined as those showing a more than 4-fold difference in expression (franking diagonal lines) in one cell type with mean log_2_ (RPM+1) ≧ 4. The genes defined as up-regulated in the PE (504 genes), the epiblast (309 genes), the pTE (231 genes) and the mTE (391 genes) are colored blue, green, yellow and orange, respectively. Key marker genes for each cell type are indicated. (**E**) The box-plot analysis of the expression levels of the genes up-regulated in mTE (left) or pTE (right) among the four cell types. The bar in the middle of the box indicates the median expression level, and the top and bottom edges of the box, and the top and bottom bars indicate the expression levels encompassing expression of 50% and ±2 SDs from the median of the genes, respectively (also in (**G**)). (**F**) Gene ontology (GO) analysis of the genes up-regulated in mTE (top) or pTE (bottom) using GO_BP_FAT collection (35). (G) The box-plot analysis of the average gene-expression levels, calculated based on the copy numbers of the ERCC spike-in RNAs, in the four cell types.

We isolated peri-implantation blastocysts at E4.5 (Supplementary Figure S5A), bisected them into embryonic (polar with ICM) and ab-embryonic (mural) parts, carefully dissociated them into single cells, picked up single cells that are considered to compose mTE or pTE/ICM and amplified their cDNAs. We examined the amplification efficiency of cDNAs by analyzing the expression level of *Gapdh* and the ERCC spike-in RNAs (Supplementary Figure S5B and C), roughly classified the cDNAs by analyzing the expression of the key markers *Nanog* (epiblast), *Gata4* (PE), *Cdx2* (TE) and *Gata2* (TE) (Supplementary Figure S5B), and among 67 single-cell cDNAs with good quality, processed 37 representative cDNAs [12 for mTE, seven for m/pTE, nine for epiblasts, nine for PE by their anatomical location and marker expression; we noted that many of the *Gata2*-positive TE lacked *Cdx2* mRNA expression at this stage, although nearly all the TE showed CDX2 protein by IF staining (Supplementary Figure S5A and B)] for the SC3-seq analysis (Supplementary Figure S5D). UHC revealed that these cells are classified largely into two clusters, both of which are further divided into two sub-clusters (Figure 5A). The one major cluster consists of two sub-clusters representing, based on the expression of key signature genes, the epiblasts and the PE (*Fgf4*, *Nanog*, *Sox2* and *Klf2* for the epiblasts and *Gata4*, *Pdgfra*, *Sox17*, *Sox7* and *Gata6* for the PE) (Figure 5B). Among the cells in the other major cluster, nine out of 12 mTE (cells we isolated from the ab-embryonic parts) were classified into a single sub-cluster and the three remaining such cells were classified into the neighboring sub-cluster, with, most likely, pTE by marker expression and anatomical location. Accordingly, pair-wise comparisons of correlation coefficients (*R*^2^) of all single cells demonstrated a close relationship between mTE and pTE (Supplementary Figure S5E). Consistent with the UHC analysis, the PCA also classified the cells into four distinct groups (Figure 5C). These findings demonstrate that the SC3-seq successfully identifies the distinct cell types present in developing embryos and that mTE and pTE exhibit/initiate differential gene expression at a global level as early as E4.5.

To further validate the power of the SC3-seq, we went on to identify genes that are differentially expressed between the epiblasts and the PE [we define the DEGs as those exhibiting differences in their average expression levels of ≧4-fold, log2 values (RPM+1) of ≧4 and FDR of <0.01 based on oneway analysis of variance (ANOVA)]. Consistent with previous findings, genes up-regulated in the epiblasts (309 genes) included *Tdgf1*, *Utf1*, *Sox2* and *Nanog* and were enriched with GO terms such as ‘regulation of transcription’, ‘negative regulation of gene expression’ and ‘negative regulation of macromolecule metabolic process’, whereas genes up-regulated in the PE (504 genes) included *Sparc*, *Lama1*, *Lamb1*, *Lamc1*, *Gata4*, *Gata6* and *Pdgfra*, and were enriched with GO terms such as ‘lipid biosynthetic process’, ‘glycerolipid metabolic process’ and ‘embryonic development ending in birth or egg hatching’ (Figure 5D and Supplementary Figure S5F and G).

We next explored the genes that are differentially expressed between pTE and mTE. There has been no systematic analysis of global gene expression of these two cell types. We identified 231 genes that are up-regulated in pTE, which include *Hspd1*, *Ddah1*, *Gsto1* and *Utf1*, and are enriched with cell cycle-related GO terms such as ‘cell cycle phase’, ‘M phase’ and ‘mitotic cell cycle’ (Figure 5D and F). We noted that the genes up-regulated in pTE in comparison to mTE were expressed in the epiblasts and the PE at similar levels to that in pTE (Figure 5E), indicating that these genes are specifically down-regulated in mTE in the blastocysts. On the other hand, we identified 391 genes that are up-regulated in mTE, which include *Slc2a3*, *Dmkn*, *Klf5*, *Gata2* and *Dppa1*, and are enriched with GO terms such as ‘vitamin transport’, ‘PDGFR signaling pathway’, ‘lipid storage’, ‘embryonic placenta development’ and ‘membrane organization’ (Figure 5D and F). These data are consistent with the idea that as early as E4.5, mTE specifically stops or slows down the mitotic cell cycle and takes on an end-replication pathway for differentiation into primary trophoblast giant cells.

We calculated the average copy numbers of the genes in each cell type and found that single mTE cells bear more abundant transcripts (∼2-fold) than single epiblast cells, PE cells and pTE cells (Figure 5G), further supporting the idea that mTE initiates an end-replication to bear a larger amount of transcripts compared to typical single cells at this embryonic stage. Thus, when used together with the ERCC spike-in RNAs, the SC3-seq allows a precise estimation of transcript levels in single cells.

#### Heterogeneity of hiPSCs under different culture conditions

**Figure 6.**
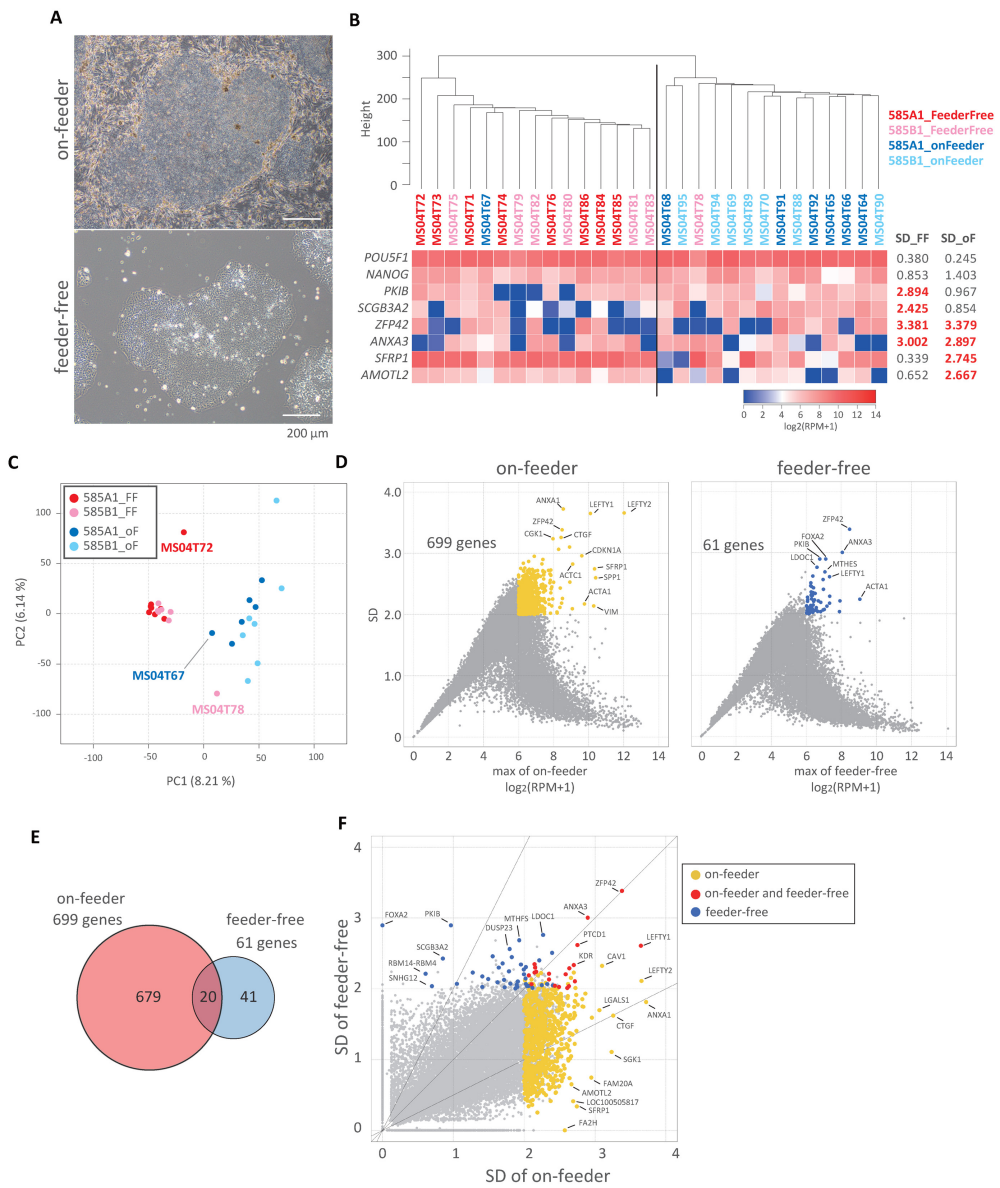
Analysis of gene expression of hiPSCs cultured with or without feeder cells. (**A**) Representative phase-contrast images of the hiPSC colonies (585B1) cultured with the SNL feeder cells (top) or without feeder free cells (bottom). Bar, 200 μm. (**B**) UHC with all expressed genes [log_2_ (RPM+1) ≧ 4 in at least one sample, 12 406 genes] (top) and heat map representation of the expression levels of genes that show highly heterogeneous expression in cells under feeder-free conditions (*PKIB* and *SCGB3A2*), under on-feeder conditions (*SFRP1* and *AMOTL2*) and under both sets of conditions (*ZFP42* and *ANXA3*). The expression levels of *POU5F1* and *NANOG* are also shown as examples of genes with small fluctuation. (**C**) PCA of all cells by all expressed genes. The cells (color code indicated) are plotted in two-dimensional space defined by PC1 and PC2. (**D**) Gene expression heterogeneity in single on-feeder and feeder-free hiPSCs analyzed by plotting the maximum expression level among the group (except the outliers, MS04T72, MS04T67 and MS04T78, as shown in (C)) against standard deviations (SDs). Genes that show the max expression level ≧ 6 and SD ≧ 2 are considered to be highly heterogeneously expressed genes [699 genes (yellow) in on-feeder hiPSCs and 61 genes (blue) in feeder-free hiPSCs]. (**E**) Venn diagram showing the relationship among the highly heterogeneously expressed genes identified in (D). (**F**) Scatter plot of the SDs of gene expression levels in on-feeder hiPSCs and in feeder-free hiPSCs. The genes are colored as in (D) and those that are highly heterogeneous under both conditions (20 genes) are colored red.

We next examined whether the SC3-seq successfully detects the heterogeneity of gene expression in seemingly homogeneous cell populations. We thus measured the gene expression of hiPSCs cultured on feeder cells (on-feeder hiPSCs) and in hiPSCs cultured under a feeder-free condition (feeder-free hiPSCs). For this purpose, we generated single-cell cDNAs of two lines of hiPSCs (585A1 and 585B1) cultured on the SNL feeder cells and cultured under a feeder-free condition (in total we generated 112 single-cell cDNAs of good quality) (Figure 6A and Supplementary Figure S6A and B) (23,25,26) and performed an SC3-seq analysis of the representative cells (seven, seven, eight and seven single cells for on-feeder 585A1, on-feeder 585B1, feeder-free 585A1 and feeder-free 585B1, respectively) (Supplementary Figure S6C). The UHC analysis revealed that the on-feeder hiPSCs and the feeder-free hiPSCs could be classified essentially into two distinct clusters irrespective of the line difference, with the exception that one on-feeder hiPSC was classified into the feeder-free cluster and one feeder-free hiPSC was classified into the on-feeder cluster (Figure 6B and Supplementary Figure S6D). This indicated the distinct, although still very similar, properties of the on-feeder and feeder-free hiPSCs. Interestingly, the PCA analysis showed that the feeder-free hiPSCs, except in the case of the two outliers, were clustered tightly together, whereas the on-feeder hiPSCs were more scattered, especially along the PC2 axis (Figure 6C), indicating that the gene expression of the on-feeder hiPSCs was more heterogeneous than that of the feeder-free hiPSCs.

To examine this point further, we plotted the SDs of the gene-expression levels against the gene-expression levels in the on-feeder and feeder-free hiPSCs. Consistent with the PCA analysis, this analysis indicated that the on-feeder hiPSCs showed higher SDs of gene-expression levels than the feeder-free iPSCs in essentially all the gene-expression ranges: we identified 699 genes that bear a maximum expression level log2 (RPM+1) ≧ 6 among the cells analyzed and the SDs ≧2 in the on-feeder iPSCs, and only 61 such genes in the feeder-free hiPSCs (Figure 6D). We then plotted the SDs of the gene-expression levels of the feeder-free iPSCs against those of the on-feeder hiPSCs and identified 41 genes with higher SDs in the feeder-free hiPSCs, including *PK1B*, *SNHG12*, *SCGB3A2*, *LDOC1*, *MTHFS* and *RBM14/RBM4*, 20 genes with high SDs (SDs ≧2) both in the feeder-free and on-feeder hiPSCs, including *ZFP42*, *ANXA3*, *LEFTY1*, *PTCD1* and *KDR*, and 679 genes with higher SDs in the on-feeder hiPSCs, including *AMOTL2*, *LEFTY2*, *ANXA1*, *SGK1*, *CTGF* and *SFRP1* (Figure 6B, E and F). Collectively, these findings demonstrate that the feeder-free hiPSCs are more homogeneous in gene expression than the on-feeder hiPSCs. We conclude that the SC3-seq is a powerful methodology that can identify the presence of heterogeneity in gene expression within seemingly homogeneous cell populations.

## DISCUSSION

We have here presented a novel single-cell RNA-seq methodology, the SC3-seq, which synthesizes, amplifies and reads the very 3-prime ends of cDNAs from single-cell RNAs, allowing highly quantitative and parallel measurements of single-cell gene expression with a relatively small sequence depth. Since the SC3-seq reads only the very 3-prime ends of mRNAs, this method is not suitable for applications that seek to identify specific splice variants in single cells. It may also be relatively difficult to discriminate allelic expression by the SC3-seq, unless there are allelic single nucleotide polymorphisms around ∼200 bp from the TTSs. However, a primary objective of single-cell gene expression analysis is to identify genes that show specific expression in relevant cells or to identify gene-expression heterogeneity among relevant cell populations. Compared to other single-cell RNA-seq methods that aim to generate, amplify and read full-length cDNAs, the SC3-seq exhibits a superior quantitative performance in that it does not underestimate the expression levels of relatively longer transcripts (Figure 4A) and it detects much larger numbers of transcripts with smaller sequence depth (Figure 4D). We therefore consider the SC3-seq a powerful strategy for such primary applications.

We provided evidence that the SC3-seq allows a highly reproducible/quantitative RNA-seq with a wide range of starting amounts of RNAs [from 100 ng (10 000-cell level) to 10 pg (1-cell level)] by applying differing numbers of amplification cycles of PCR for the preparation of initial cDNAs (Figure 2). Indeed, the reproducibility of the SC3-seq for the starting materials from 10 000 cells to as little as 10 cells is extremely high (*R*^2^ ≧ 0.940) (Figure 2). The SC3-seq is therefore a versatile methodology suitable not only for single-cell gene expression analysis but also for analysis of the gene expression in materials prepared from a relatively large number of cells with similar properties, such as the developing epiblasts or cell populations expressing common markers sorted by fluorescent-activated cell sorting.

We consider that the decline in the correlation for the SC3-seq with 1-cell level RNAs (10 000-cell level versus 1-cell level: *R*^2^ = 0.776–0.797; 1-cell level versus 1-cell level: *R*^2^ = 0.677–708) (Figure 2), although still a good correlation, may have been partly attributable to a stochastic loss of RNAs with low expression levels during the preparation of the dilution samples. Our reasoning is as follows. First, the correlation is higher for RNAs expressed at substantial levels (e.g. more than ∼20 copies/10-pg RNAs) (Figure 2). Second, the correlation coefficient between the SC3-seq for 10 000 cell-level RNAs and for 1-cell level RNAs (*R*^2^ = 0.776–0.797) is substantially higher than that between two independent SC3-seq analyses for 1-cell level RNAs (*R*^2^ = 0.677–708). Indeed, we have evidence that amplification by SC3-seq from 10-pg RNAs of genes expressed at low levels (e.g. less than ∼20 copies/10-pg RNAs) results either in representative or no amplification, the latter being presumably mainly due to the loss of transcripts during dilution (data not shown). We therefore propose that the SC3-seq is a highly representative protocol for single-cell gene expression analysis.

Accordingly, we applied the SC3-seq to address two biological issues: decomposition of the cell types in the E4.5 mouse blastocysts and identification of cellular heterogeneity of hiPSCs cultured under different conditions (Figures 5 and 6). For the former issue, we successfully identified four distinct cell types that comprise the blastocysts and their global gene-expression differences. The identification of genes that are up-regulated in E4.5 epiblasts in comparison to the PE will be critical to understand the mechanism for epiblast formation, a primed state for differentiation into three germ layers. Furthermore, to our knowledge, the present work is the first to identify the global gene-expression differences between the pTE, precursors for the extraembryonic ectoderm and the mTE, precursors for the primary trophoblast giant cells. Our analysis revealed that the mTE exhibited a gene expression profile indicative of the cessation/slow-down of the mitotic cell cycle and of differentiation into primary trophoblast giant cells (42) and a larger amount of total transcript levels in comparison to the other three cell types in the blastocysts, indicating that the E4.5 mTE cells are starting differentiation into primary trophoblast giant cells with their characteristic end-replication cycles. Our study thus also provides a foundation for understanding the mechanism of the formation of primary trophoblast giant cells.

With respect to the latter issue, remarkably, we showed that the single on-feeder hiPSCs exhibited much higher heterogeneity in gene expression than the single feeder-free hiPSCs: we identified 699 genes that expressed log2 (RPM+1) values of ≧6 (maximum level among the cells analyzed) and bore SDs of gene expression levels among different single cells of ≧2 in the on-feeder hiPSCs, whereas we detected only 61 such genes in the feeder-free hiPSCs (Figure 6). Since the feeder-free hiPSCs are less heterogeneous, which would be better for homogeneous differentiation, our findings indicate they are not only useful in culture, but would also be a superior starting material for a more uniform differentiation toward lineages of interest. Notably, under both conditions, *ZFP42*, a gene known to be expressed in naïve but not primed epiblast cells in mice and to show heterogeneity in mESCs cultured with serum (43), was one of the most heterogeneously expressed genes. Considering that *ZFP42* is expressed at a high level in the ICM of the human pre-implantation blastocysts (*ZFP42* is also expressed in TE and PE in humans) (34), this finding may indicate that hiPSCs are, to some extent, fluctuating between a naïve and a primed state. Further studies, including those clarifying the functions of the relevant genes, are necessary to more precisely determine the properties of hiPSCs/ESCs. In conclusion, we have established the SC3-seq and demonstrated its capacity to address biologically important questions. Based on our findings, we propose that SC3-seq is one of the most practical approaches for single-cell gene expression analysis in biologically relevant contexts.

## ACCESSION NUMBERS

Accession numbers for the data generated in this study and for the published data used in this study are as follows: the SC3-seq data (GSE63266), the RNA-seq data for mESCs and mouse embryonic fibroblasts (MEFs) (GSE45916) (33), Smart-seq2 data for MEF (GSE49321) (13) and the single-cell RNA-seq data for hESCs by Yan *et al*. (GSE36552) (34).

## Supplementary Material

Supplementary Data
