# Supplementary material for: SC3-seq: a method for highly parallel and quantitative measurement of single-cell gene expression
Source: Nucleic Acids Res. 2015 Feb 26;43(9):e60. doi: 10.1093/nar/gkv134 (PMC4482058; doi:10.1093/nar/gkv134)
Supplement: Supplementary Data [file gkv134_suppl.zip › nar-03162-met-g-2014-File008.pdf]

SUPPLEMENTARY MATERALS

SUPPLEMENTAL TABLES, see separate Excel documents

Supplementary Table S1. Summary of the mapping statistics of the SC3-seq sequence reads

Supplementary Table S2. Copy numbers of the ERCC spike-in RNAs provided per 10 pg of total RNAs or in single-cell samples

Supplementary Table S3. Oligonucleotides/primers used in this study

Nakamura et al.
Figure S1

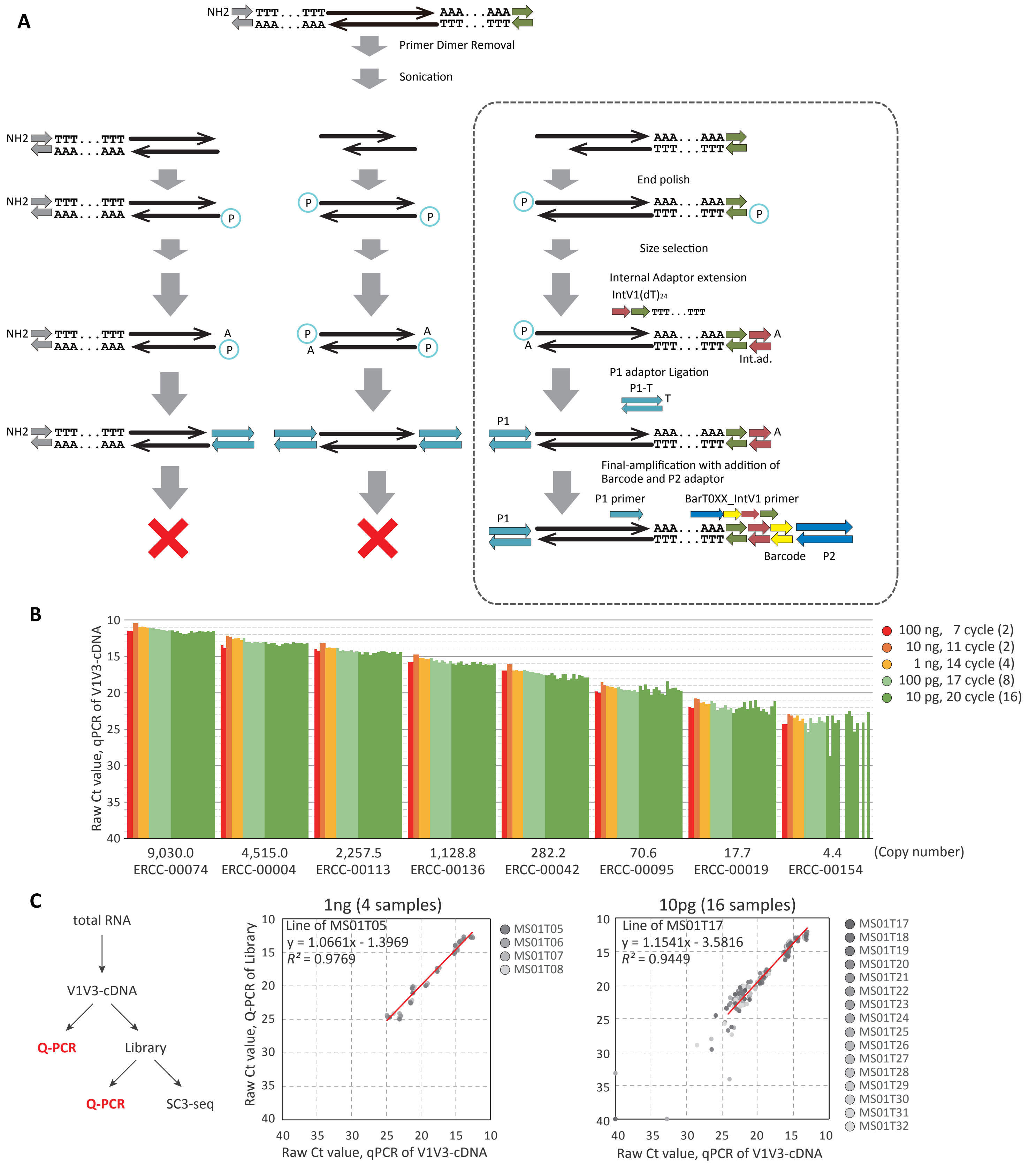

Supplementary Figure S1. Establishment of SC3-seq, related to Figure 1

(A) Mechanism for the enrichment of the 3-prime ends (V1-tagged ends) of the cDNAs by the library construction step of the SC3-seq. After fragmentation of the amplified cDNAs by sonication, there should be three types of fragments in a tube, the fragments bearing the V3-tagged ends (the 5-prime ends), the fragments without the tags (inner fragments), and the fragments bearing the V1-tagged ends. All of them are polished and phosphorylated in the end-polish step. In the Internal adaptor extension step, only the fragments bearing the 3-prime ends of the cDNAs with the V1-tagged sequence are captured and provided with the internal adaptor sequence, while the others are not. In the P1 adaptor ligation step, although the fragments bearing the V3-tagged ends and the inner fragments are potentially jointed with the P1 adaptor, only the opposite sides of the V1-tags of the V1-end fragments are successfully ligated with the P1 adaptor, because the V1-ends are not phosphorylated. Consequently, the fragments harboring both the P1 and the Int-V1 tags are enriched in the final amplification step.

(B) Q-PCR analysis of the amplification levels of the ERCC spike-in RNAs in amplified cDNAs from 100 ng, 10 ng, 1 ng, 100 pg, and 10 pg of ESC total RNAs (color codes indicated). The copy numbers per 10 pg RNAs and the corresponding ERCC codes are shown.

(C) Comparison of the expression levels of the genes listed in Table S3 in amplified cDNAs [V1V3 cDNAs, from 1 ng (left, 4 samples) and 10 pg (right, 16 samples) total RNAs] with those in the SC3-seq library by Q-PCR (raw CT value). Regression lines of representative samples are shown (MS01T05 and MS01T17 for 1 ng and 10 pg, respectively, of total RNAs).

Nakamura et al.
Figure S2

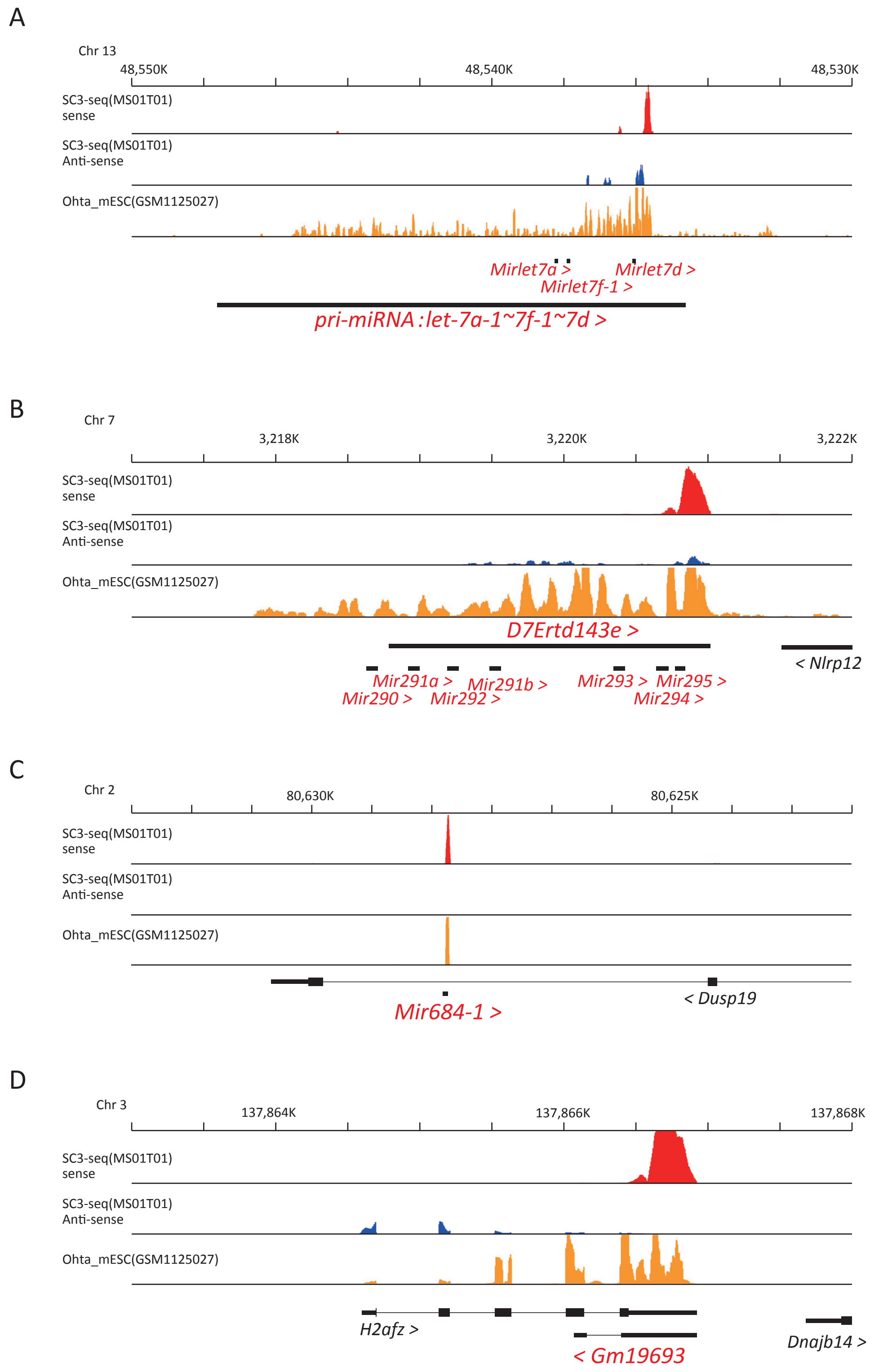

Supplementary Figure S2. Examples of the SC3-seq reads at non-coding RNA loci, related to Figure 1

(A) The *Let7a-7d* locus. In (A-D), the red peaks indicate the reads mapped on the sense strands; the blue peaks show the reads mapped on the anti-sense strands. The orange peaks are the reads mapped by Ohta et al. (1). The 3-prime end of primary (pri) miRNA is detected by the SC3-seq. The annotations for pri-miRNAs are based on (2).

(B) The *Mir290-295* locus. The noncoding transcript *D7Ertd143e* is presumably the primary precursor for *Mir290-295*.

(C) The *Mir684-1* locus. A single miRNA is encoded in an intron of the coding gene *Dusp19*. The SC3-seq reads were perfectly overlaped with those by (1), suggesting that a short poly (A)-tailed miRNA precursor is transcribed from the locus.

(D) An unclassified non-coding RNA, *Gm19693*, is annotated at the reverse strand of the 3-prime end of the *H2afz* transcript. Since up to 10% of the SC3-seq reads were mapped at the opposite strands in a broader manner (Figure 1D), a small fraction of the anti-sense reads of *H2afz* were assigned to *Gm19693*. In (A-D), symbols for coding and non-coding transcripts are shown in black and red, respectively.

Nakamura et al.

Figure S3

A

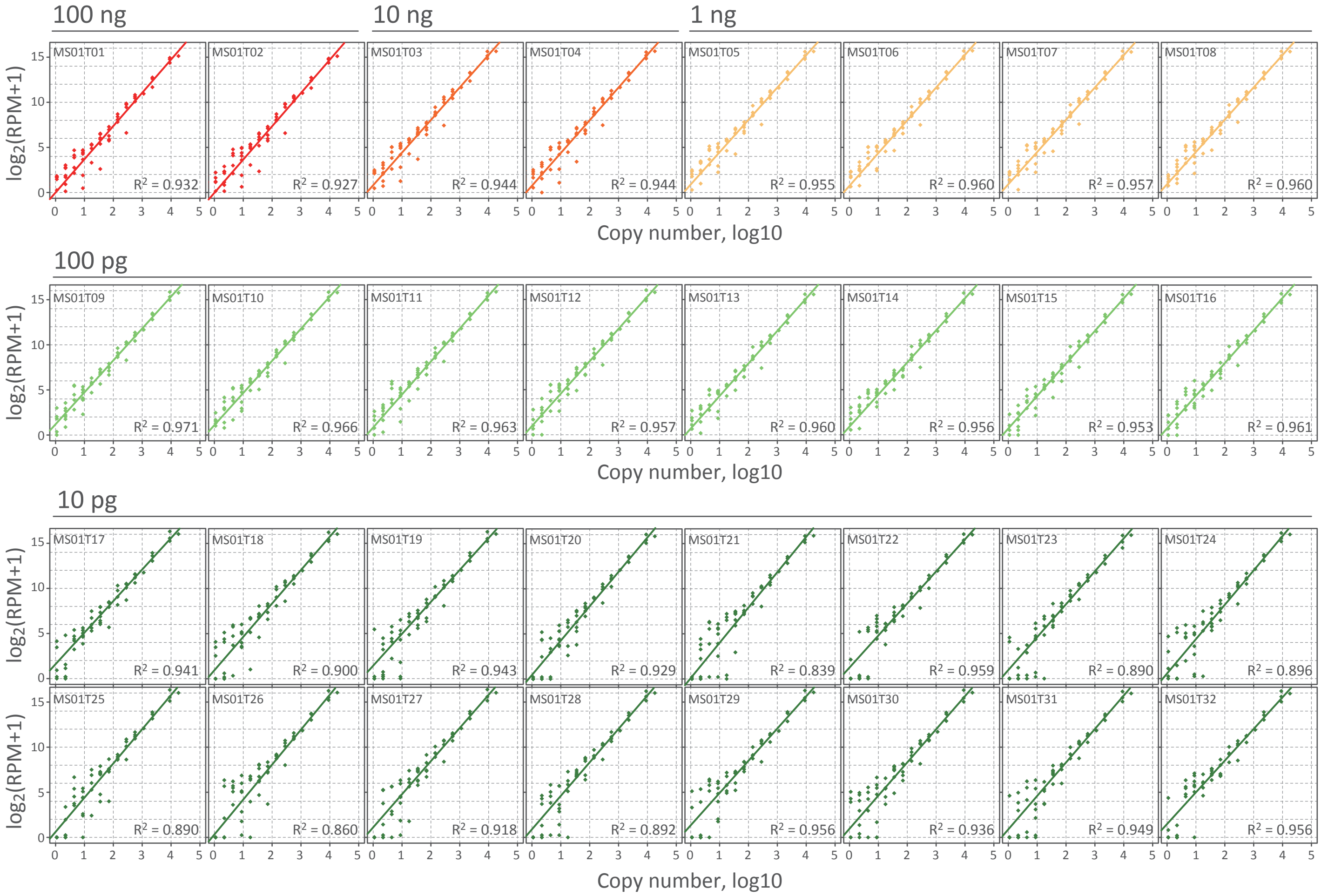

B

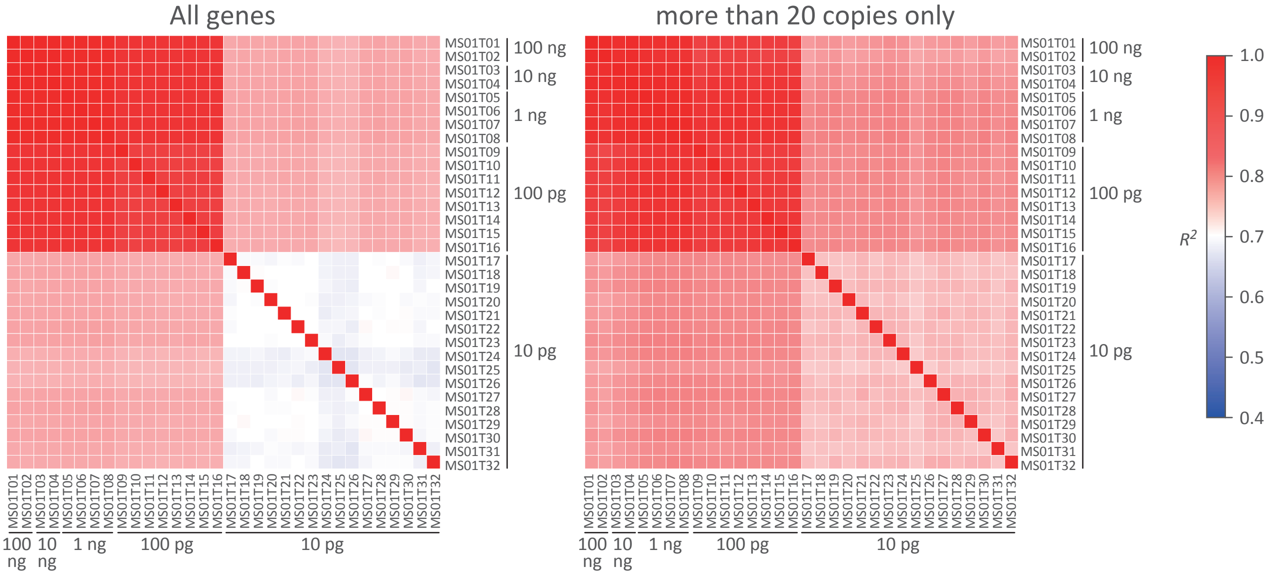

C

All genes

	100ng	10ng	1ng	100pg	10pg
100ng	0.995			R^2 value	max<br>min
10ng	0.993<br>0.991	0.992			
1ng	0.989<br>0.987	0.990<br>0.988	0.988<br>0.988		
100pg	0.964<br>0.961	0.966<br>0.962	0.965<br>0.962	0.945<br>0.940	
10pg	0.797<br>0.776	0.800<br>0.777	0.802<br>0.780	0.794<br>0.770	0.708<br>0.677

more than 20 copies only

	100ng	10ng	1ng	100pg	10pg
100ng	0.933			R^2 value	max<br>min
10ng	0.985<br>0.978	0.989			
1ng	0.976<br>0.966	0.987<br>0.974	0.991<br>0.989		
100pg	0.950<br>0.930	0.961<br>0.939	0.970<br>0.959	0.958<br>0.947	
10pg	0.825<br>0.789	0.837<br>0.801	0.850<br>0.818	0.844<br>0.814	0.786<br>0.747

Supplementary Figure S3. Quantitative performance of the SC3-seq, related to Figure 2

(A) Correlations between the quantities of the ERCC RNAs spiked in a dilution series of mESC total RNAs (100 ng: two replicates; 10 ng: two replicates; 1 ng: four replicates; 100 pg: eight replicates; 10 pg: sixteen replicates) and the estimated levels of the spike-in RNAs by the SC3-seq [$\log_2$ (RPM+1)]. The SC3-seq data for the ERCC spike-in RNAs with more than 10 copies per 10 pg are used for the regression line.

(B) Heat-map representation of the correlation coefficients (R^2) among all samples amplified and measured by the SC3-seq from a dilution series of ESC total RNAs (left: all expression ranges; right: genes expressing more than 20 copies per 10 pg of RNAs).

(C) Minimum (min) and maximum (max) values of correlation coefficients (R^2) in the pair-wise comparison groups shown in (B) (left: all expression ranges; right: genes expressing more than 20 copies per 10 pg of RNAs).

Nakamura et al.
Figure S4

A

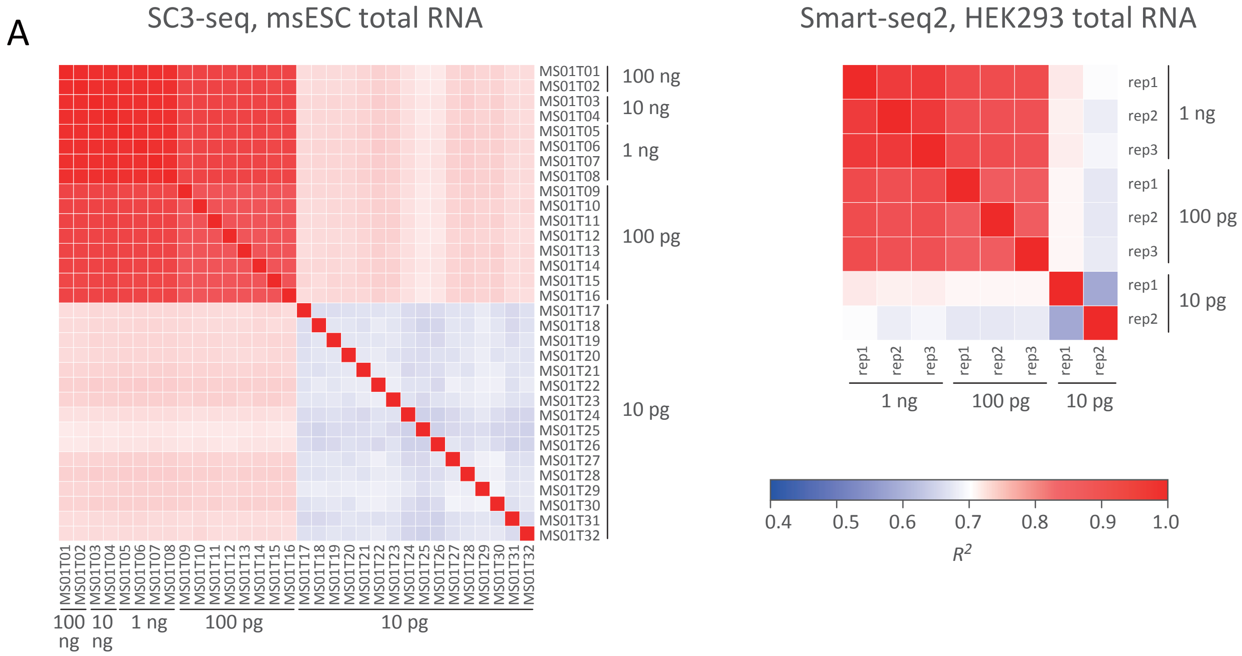

B

SC3-seq, msESC total RNA

R^2 value

	100ng	10ng	1ng	100pg	10pg
100ng	0.985				max<br>min
10ng	0.983<br>0.981	0.982			
1ng	0.976<br>0.975	0.977<br>0.955	0.972<br>0.972		
100pg	0.938<br>0.933	0.939<br>0.935	0.937<br>0.932	0.914<br>0.908	
10pg	0.744<br>0.718	0.747<br>0.721	0.750<br>0.722	0.746<br>0.718	0.689<br>0.659

Smart-seq2, HEK293 total RNA

R^2 value

	1ng	100pg	10pg
1ng	0.955<br>0.951		max<br>min
100pg	0.927<br>0.916	0.901<br>0.898	
10pg	0.721<br>0.685	0.709<br>0.680	0.613

C

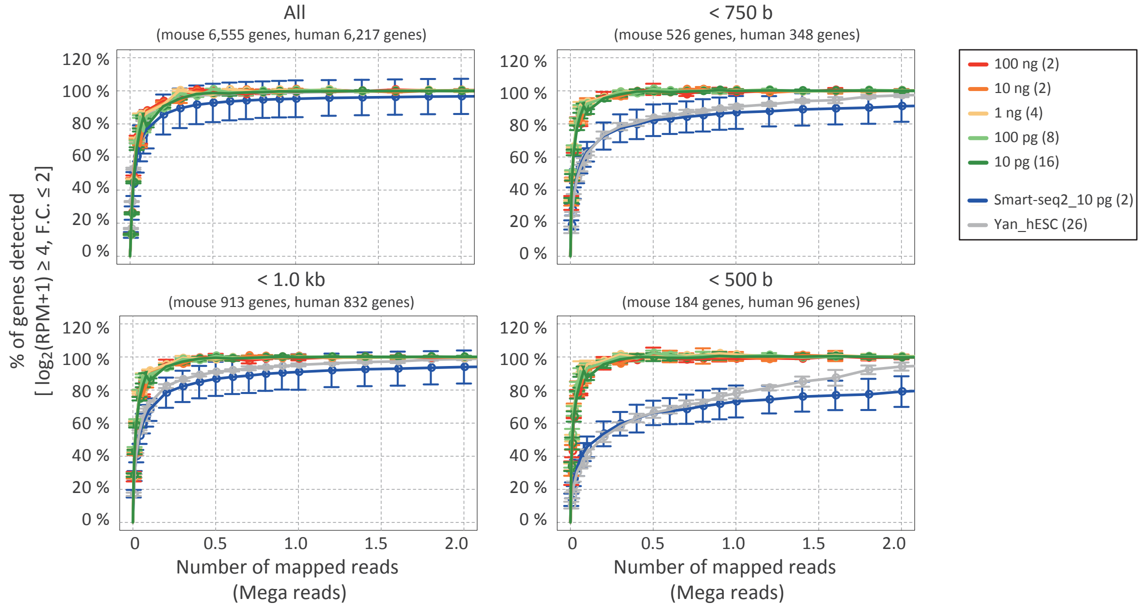

Supplementary Figure S4. Comparison of the performance of the SC3-seq with that of other single-cell RNA-seq methods, related to Figure 4

(A) Heat-map representation of the correlation coefficients (R^2) among dilution samples amplified and measured by the SC3-seq (left) and the Smart-seq2 (right). Note that the expression values less than 0.1 RPM and 0.1 FPKM by the SC3-seq and the Smart-seq2, respectively, were set as 0.1 according to (3).

(B) Minimum (min) and maximum (max) values of correlation coefficients (R^2) in pair-wise comparison groups shown in (A) (left: SC3-seq; right: Smart-seq2).

(C) Analysis for saturation of detection [gene-expression levels ≧ the top 6,555th and 6,217th genes for mice and humans, respectively (1/4 of all the annotated transcripts for mice and humans), ~$\log_2$ RPM ≧ 3.69±0.05 (SC3-seq), ~$\log_2$ FPKM ≧ 2.21±1.28 (Yan et al.), and ~$\log_2$ FPKM ≧ 2.92±0.27 (Picelli et al.), fold changes of gene expression levels ≦ 2 in comparison to those determined by the full sequence reads [Table S1 or (3,4)]] for all (top) and < 1 Kbp, < 750 bp and < 500 bp transcripts by the three single-cell RNA-seq methodologies.

Nakamura et al.

Figure S5

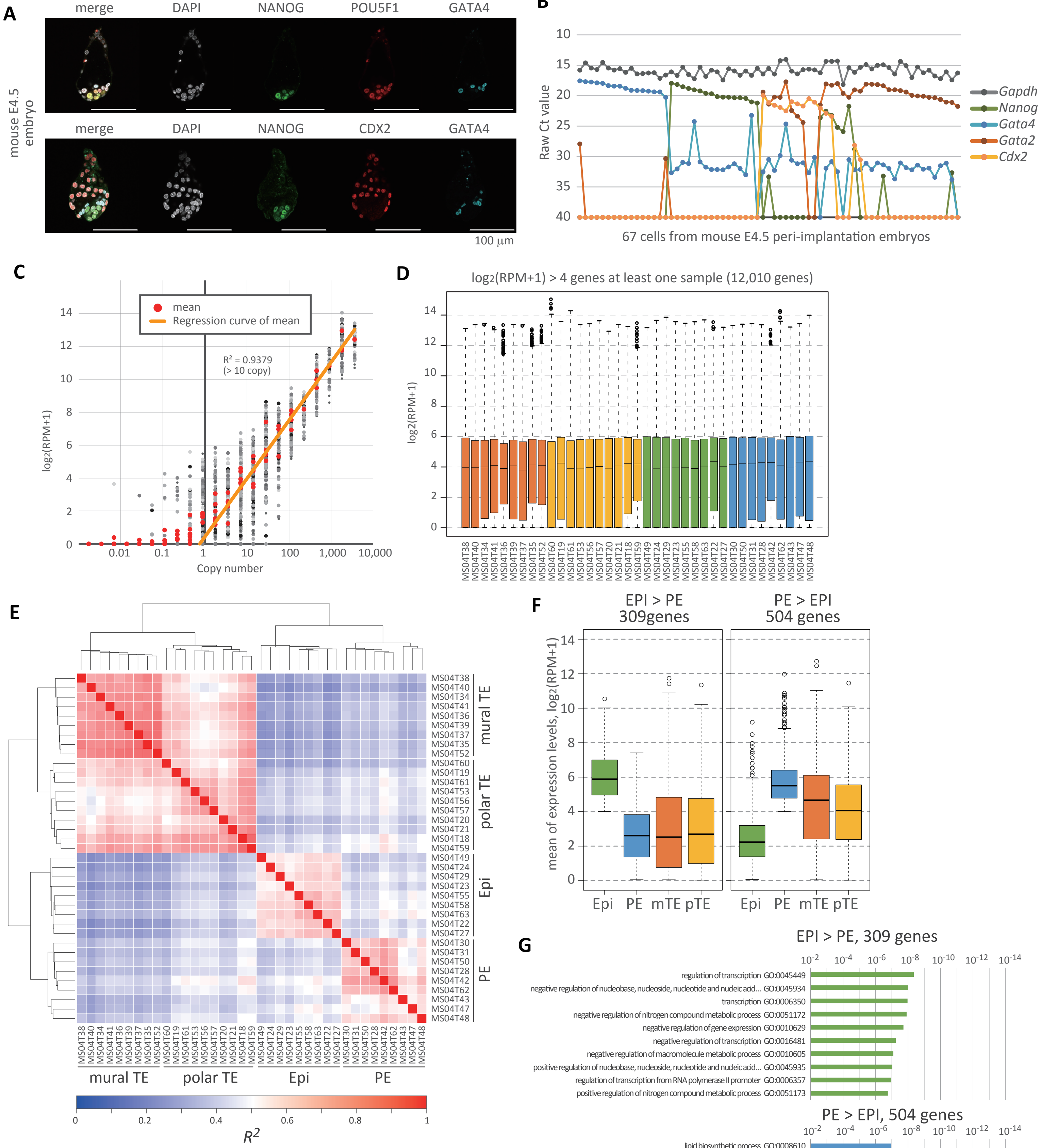

Supplementary Figure S5. Analysis of mouse E4.5 blastocysts, related to Figure 5

(A) Immunofluorescence analysis of the expression of key lineage markers [NANOG (epiblasts), POU5F1 (epiblasts and PE), GATA4 (PE), and CDX2 (TE)] in the mouse E4.5 peri-implantation embryos. Scale bar = 100 µm.

(B) Q-PCR analysis of the expression of key lineage markers [Nanog (epiblasts), Gata4 (PE), Gata2 (TE), Cdx2 (TE), Gapdh (housekeeping), color code indicated] in the amplified cDNAs (quality-checked 67 cDNAs) from single cells of E4.5 embryos. Samples are aligned by the order of the expression levels of the indicated marker genes.

(C) Scatter-plot comparison of the ERCC spike-in RNA levels in amplified samples measured by the SC3-seq [$\log_2$ (RPM+1)] with their original copy numbers. The regression curve and correlation coefficient were calculated from the mean of probes whose copy numbers are more than 10.

(D) The box-plot analysis of the distribution of the gene-expression levels in each cell. The bar in the middle of the box indicates the median expression level, and the top and the bottom edges of the box, and the top and the bottom bars indicate the expression levels encompassing expression of 50% and ±2 SDs from the median of the genes, respectively.

(E) Heat-map representation of the correlation coefficients (R^2) among all single embryonic cells.

(F) The box-plot analysis of the expression levels of the genes up-regulated in the epiblasts compared to the PE (left) or those up-regulated in the PE compared to the epiblasts (right), as indicated in Figure 5D.

(G) GO analysis of the genes up-regulated in the epiblasts compared to the PE (top) or those up-regulated in the PE compared to the epiblasts (bottom) using GO_BP_FAT collection.

Nakamura et al.
Figure S6

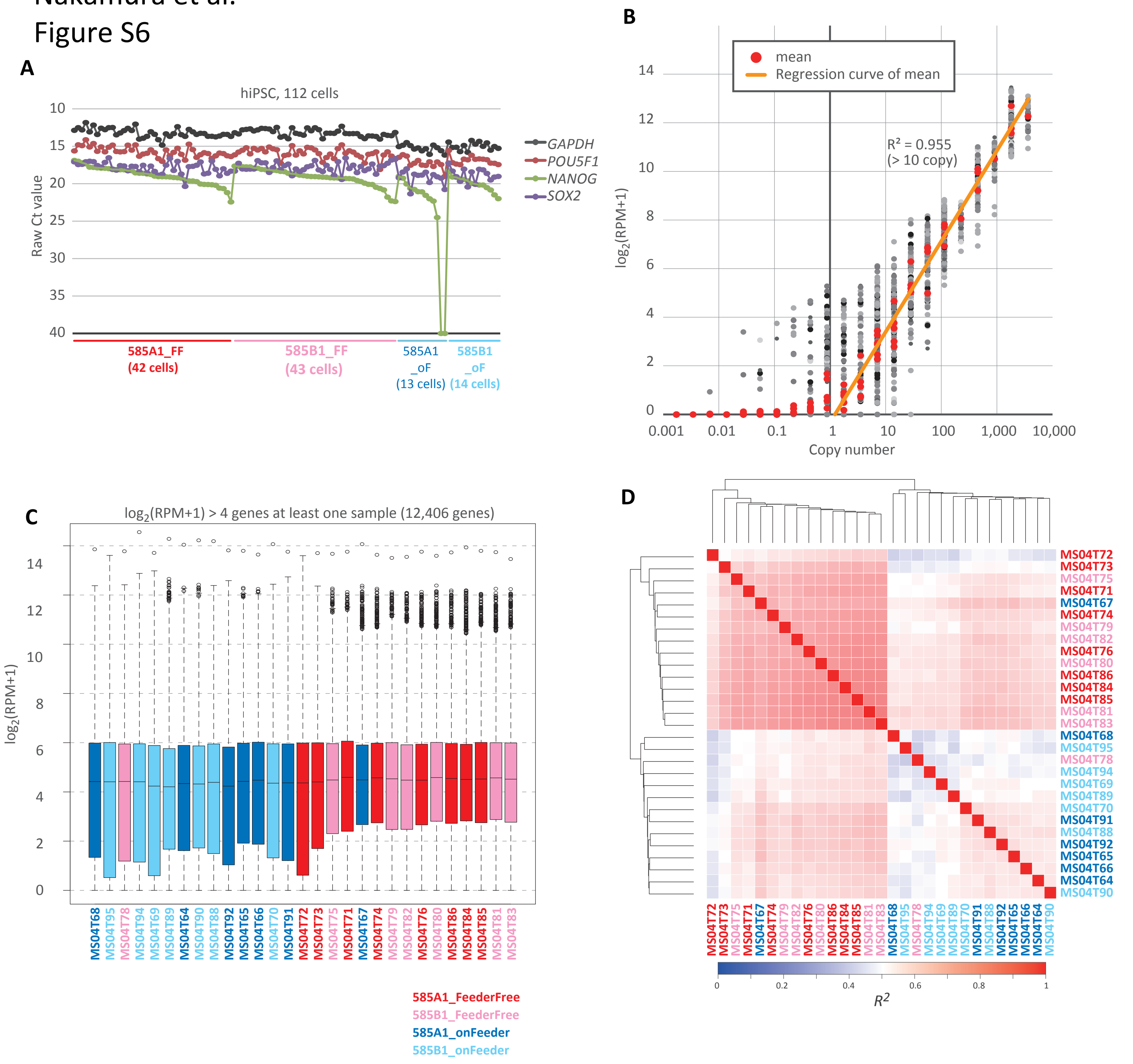

Supplementary Figure S6. Analysis of gene expression of hiPSCs cultured with or without feeder cells, related to Figure 6

(A) Q-PCR analysis of the expression of key pluripotency genes [*POU5F1, NANOG, SOX2,* and *GAPDH* (housekeeping), color code indicated] in the amplified cDNAs (112 quality-checked cDNAs) from hiPSCs (585A1 and 585B1) cultured with or without feeder cells. Samples are aligned by the order of the culture condition, cell lines, and the expression levels of *NANOG*.

(B) Scatter-plot comparison of the ERCC spike-in RNA levels in amplified samples measured by the SC3-seq [$\log_2$ (RPM+1)] with their original copy numbers. The regression curve and correlation coefficient were calculated from the mean of probes whose copy numbers are more than 10.

(C) The box-plot analysis of the distribution of the gene-expression levels in each cell. The bar in the middle of the box indicates the median expression level, and the top and the bottom edges of the box, and the top and the bottom bars indicate the expression levels encompassing expression of 50% and ±2 SDs from the median of the genes, respectively.

(D) Heat-map representation of the correlation coefficients (R^2) among all single hiPSCs.

REFERENCES

1. Ohta, S., Nishida, E., Yamanaka, S. and Yamamoto, T. (2013) Global splicing pattern reversion during somatic cell reprogramming. *Cell reports*, **5**, 357-366.
2. Saini, H.K., Enright, A.J. and Griffiths-Jones, S. (2008) Annotation of mammalian primary microRNAs. *BMC Genomics*, **9**, 564.
3. Picelli, S., Bjorklund, A.K., Faridani, O.R., Sagasser, S., Winberg, G. and Sandberg, R. (2013) Smart-seq2 for sensitive full-length transcriptome profiling in single cells. *Nat Methods*, **10**, 1096-1098.
4. Yan, L., Yang, M., Guo, H., Yang, L., Wu, J., Li, R., Liu, P., Lian, Y., Zheng, X., Yan, J. *et al.* (2013) Single-cell RNA-Seq profiling of human preimplantation embryos and embryonic stem cells. *Nat Struct Mol Biol*, **20**, 1131-1139.
